# Biallelic mutations in the death domain of *PIDD1* impair caspase-2 activation and are associated with intellectual disability

**DOI:** 10.1038/s41398-020-01158-w

**Published:** 2021-01-05

**Authors:** Taimoor I. Sheikh, Nasim Vasli, Stephen Pastore, Kimia Kharizi, Ricardo Harripaul, Zohreh Fattahi, Shruti Pande, Farooq Naeem, Abrar Hussain, Asif Mir, Omar Islam, Katta Mohan Girisha, Muhammad Irfan, Muhammad Ayub, Christoph Schwarzer, Hossein Najmabadi, Anju Shukla, Valentina C. Sladky, Vincent Zoran Braun, Irmina Garcia-Carpio, Andreas Villunger, John B. Vincent

**Affiliations:** 1 Molecular Neuropsychiatry & Development (MiND) Lab, Campbell Family Mental Health Research Institute, Centre for Addiction and Mental Health, Toronto, ON M5T 1R8 Canada; 2 Institute of Medical Science, University of Toronto, Toronto, ON Canada; 3 Molecular Genetics Laboratory, North York General Hosptial Genetics Program, Toronto, ON M2K 1E1 Canada; 4 Genetics Research Center, University of Social Welfare and Rehabilitation Sciences, Tehran, 19834 Iran; 5 Department of Medical Genetics, Kasturba Medical College, Manipal, Manipal Academy of Higher Education, Manipal, Karnataka 576104 India; 6 General and Health Systems Psychiatry, Centre for Addiction and Mental Health, Toronto, ON M5T 1R8 Canada; 7 Department of Psychiatry, University of Toronto, Toronto, ON M5T 1R8 Canada; 8 Human Molecular Genetics Lab, Department of Biological Sciences, FBAS, International Islamic University, Islamabad, Pakistan; 9 Department of Diagnostic Radiology, Queens University, Kingston, ON K7L 2V7 Canada; 10 Department of Mental Health, Psychiatry and Behavioural Sciences, Peshawar Medical College, Riphah International University, Islamabad, Pakistan; 11 Lahore Institute of Research & Development, Lahore, 51000 Pakistan; 12 Department of Psychiatry, Queen’s University, Kingston, ON K7L 3N6 Canada; 13 Department of Pharmacology, Medical University of Innsbruck, Innsbruck, Austria; 14 Kariminejad-Najmabadi Pathology and Genetics Center, Tehran, 14667 Iran; 15 Institute for Developmental Immunology, Biocenter Medical University of Innsbruck, Innsbruck, Austria; 16 CeMM Research Center for Molecular Medicine of the Austrian Academy of Sciences, Vienna, Austria; 17 Ludwig Boltzmann Institute for Rare and Undiagnosed Diseases, Vienna, Austria

**Keywords:** Genetics, Neuroscience

## Abstract

*PIDD1* encodes p53-Induced Death Domain protein 1, which acts as a sensor surveilling centrosome numbers and p53 activity in mammalian cells. Early results also suggest a role in DNA damage response where PIDD1 may act as a cell-fate switch, through interaction with RIP1 and NEMO/IKKg, activating NF-κB signaling for survival, or as an apoptosis-inducing protein by activating caspase-2. Biallelic truncating mutations in CRADD—the protein bridging PIDD1 and caspase-2—have been reported in intellectual disability (ID), and in a form of lissencephaly. Here, we identified five families with ID from Iran, Pakistan, and India, with four different biallelic mutations in *PIDD1*, all disrupting the Death Domain (DD), through which PIDD1 interacts with CRADD or RIP1. Nonsense mutations Gln863* and Arg637* directly disrupt the DD, as does a missense mutation, Arg815Trp. A homozygous splice mutation in the fifth family is predicted to disrupt splicing upstream of the DD, as confirmed using an exon trap. In HEK293 cells, we show that both Gln863* and Arg815Trp mutants fail to co-localize with CRADD, leading to its aggregation and mis-localization, and fail to co-precipitate CRADD. Using genome-edited cell lines, we show that these three *PIDD1* mutations all cause loss of PIDDosome function. *Pidd1* null mice show decreased anxiety, but no motor abnormalities. Together this indicates that *PIDD1* mutations in humans may cause ID (and possibly lissencephaly) either through gain of function or secondarily, due to altered scaffolding properties, while complete loss of PIDD1, as modeled in mice, may be well tolerated or is compensated for.

## Introduction

Biallelic missense mutations in the gene encoding caspase and RIP adaptor with death domain, CRADD (also known as RAIDD), have been reported to cause intellectual disability (ID) with enlarged head circumference (MRT34; MIM 614499; ref. ^1^). Mild lissencephaly has also been reported in affected individuals^2–6^, and megalencephaly without obvious cortical lamination defects in *Cradd* null mice^2^. Together with PIDD1 (p53-Induced Death Domain protein 1), CRADD can activate caspase-2—an endopeptidase thought to regulate apoptosis upon DNA damage in the so-called PIDDosome complex [see ref. ^7^ reviewed in refs. ^8,9^]. However, recent studies suggest a crucial role in p53 activation upon centrosome accumulation^10,11^. Alternatively, PIDD1 can also interact with RIP1 and NEMO upon DNA damage and promote nuclear factor κB (NF-κB) activation for cell survival and sterile inflammation^12,13^. Several reports implicate caspase-2 in neuronal cell death caused by NGF or beta-amyloids^8,14^. Whether these effects of caspase-2 require PIDDosome formation remains less clear^15^. Caspase-2 can also process tau protein leading to its accumulation in dendritic spines, which have been found to be increased in cognitively impaired mice and humans^16^. Moreover, loss of caspase-2 associates with increased anxiety behavior, but not memory loss in mice^17^. Together, these studies indicate that caspase-2 activity controls neuronal stress responses and cognitive brain function. As PIDDosome-independent activation of caspase-2 has been reported in hematopoietic cells, fibroblasts, and neurons^14,18^, it remains unclear if PIDD1 is needed for normal brain function.

**Fig. 1:**
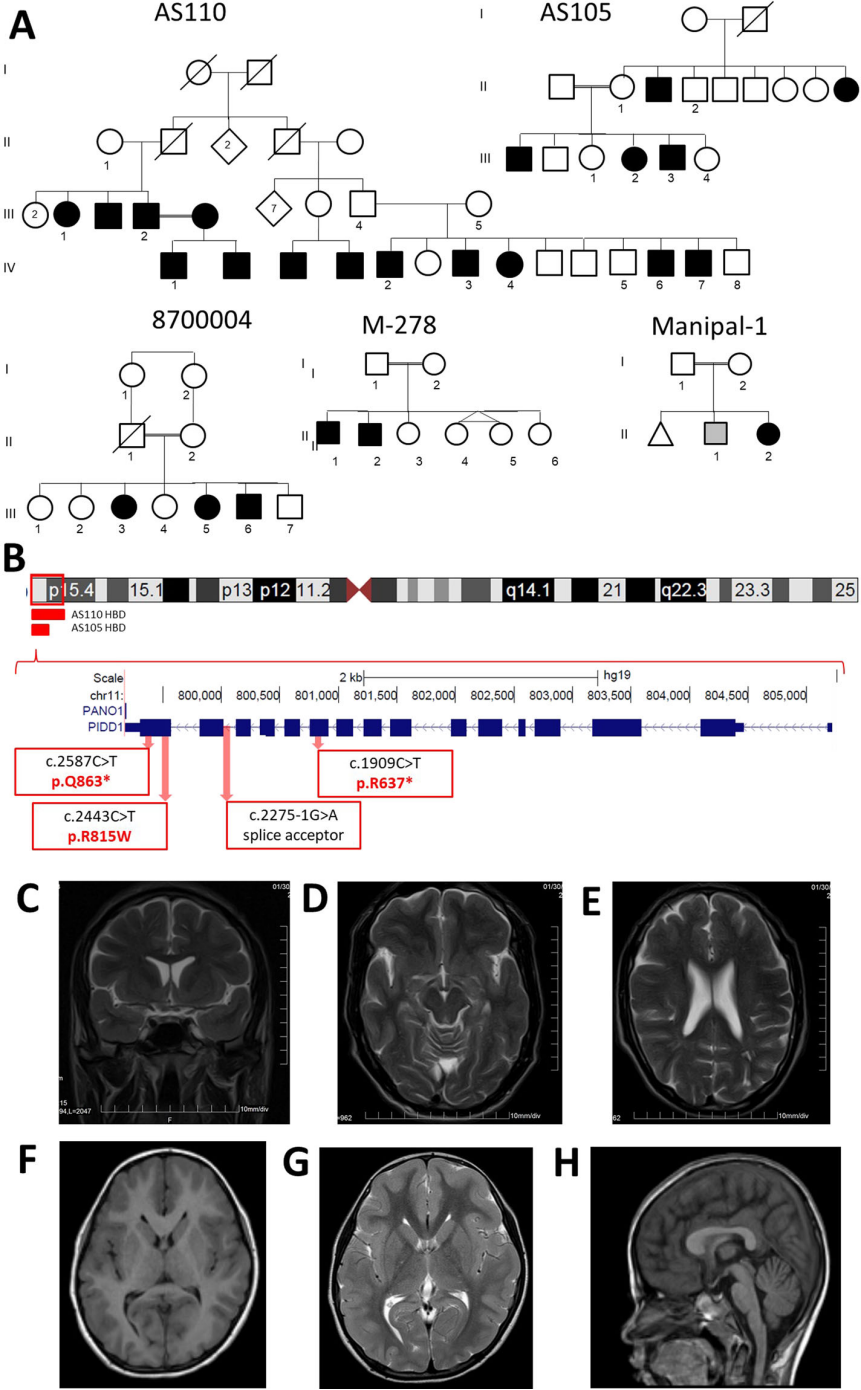
*PIDD1* mutation families. **A** Pedigrees of five families with *PIDD1* mutations. Pakistani families AS105 and AS110 were reported in Harripaul et al. (2018). Iranian families 8700004 and M278 have also been reported in Hu et al (2019). Indian family Manipal-1 has not been reported previously. **B** Location and overlap of HBD regions, and locations of the identified mutations relative to the exonic structure of *PIDD1*. The known PIDD1 protein cleavage products, N-PIDD1, C-PIDD1, and CC-PIDD1 are shown, as reported by Tinel et al. (2007). MRI images for AS110 IV-3. **C** Coronal T2 sequence shows subtle smooth gyration and shallow sulcation affecting the frontal and temporal lobes, compatible with lissencephaly. **D** Axial T2 sequence shows subtle smooth gyration and shallow sulcation affecting the frontal and temporal lobes, compatible with lissencephaly. **E** Axial T2 sequence shows subtle smooth gyration and shallow sulcation affecting the frontal lobes, compatible with lissencephaly. MRI of the Manipal-1 proband (II-3) showing pachygyria in **F** T1 axial, **G** T2 axial, and **H** sagittal section showing mildly dysmorphic (short and thick) corpus callosum.

**Fig. 2:**
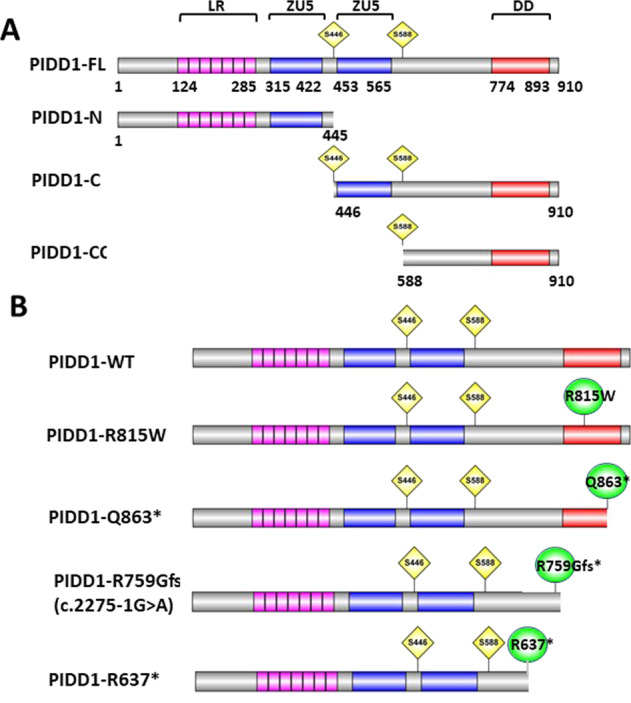
Domain structure and autoprocessing of PIDD1 protein, relative to reported mutations. **A** Constitutive processing of PIDD gives rise to PIDD1-N (~48 kDa) and PIDD1-C (~51kDa). PIDD1-N contain a leucine-rich (LR) domain while PIDD1C contain a death domain between residues 774 to 893. S446 (generating PIDD1-C) and S588 (generating PIDD1-CC) are involved in this autoprocessing mechanism. PIDD1-C later undergoes further auto-cleavage resulting in the PIDD1-CC (~37 kDa) peptide. **B** Location of the mutations identified (p.R815W and p.Q863*) within the death domain of PIDD1 protein, and the prediction based on the exon-trap results for the effect of c.2275-1G>A, namely p.R759Gfs*1.

Recently, we reported the identification of a biallelic nonsense mutation (Chr11:799453G>A; NM_145886.3:c.2587C>T; p.Gln863*) within the *PIDD1* gene in two Pakistani families segregating with non-syndromic ID^19^. PLINK: pair-wise PI_HAT values between members of the two families <0.1, suggested that the two families are not closely related. Linkage analysis for the two families gave a LOD score of 3.7. The variant is not present in the Greater Middle East Variome (GMEV)^20^ dataset, and in the gnomAD dataset (v2.1.1; gnomad.broadinstitute.org) of 246,378 chromosomes, a single (heterozygous) occurrence is reported (allele frequency = 4E−6). The mutation is predicted to truncate the protein midway through the Death Domain (DD). As the mutation occurs within the last exon, nonsense-mediated mRNA decay is not predicted, and it is anticipated that the etio-pathological mechanism is through the loss of function of the DD, but this has not been formally tested. Three additional ID families with biallelic *PIDD1* mutations have since been identified, including the missense mutation chr11:799846G>A; NM_145886.3: c.2443C>T; p.Arg815Trp (2 (heterozygous) out of 266,508 alleles in gnomAD; MAF = 7.5E−6), a splice acceptor site mutation chr11: 800015C>T; NM_145886.3: c.2275-1G>A (not present in gnomAD)^21^, and a nonsense mutation chr11:800770>A; NM_145886.3: c.1909C>T; p.Arg637* (4 (heterozygous) out of 152,564 alleles in gnomAD; MAF = 2.6E−5). The Arg815Trp mutation, also within the DD, is predicted to be pathogenic, as is the splice variant. Pedigrees and mapping information are shown in Fig. 1. The constitutive processing of PIDD1 protein is demonstrated in Fig. 2, indicating the position and effect of the reported mutations relative to the cleaved PIDD1 and its known functional domains.

Using cell biological assays we demonstrate that all these mutations act as hypomorphs, severely affecting the ability of PIDD1 to activate p53 in response to extra centrosomes. Behavioral studies in mice do support a role for the PIDDosome in anxiety behavior but did not reveal cognitive deficits. We conclude that in mice loss of PIDDosome function is well tolerated or compensated for, while truncating mutations found in humans may lead to additional effects, related to the scaffolding function of PIDD1 in multiprotein complexes, such as those affecting inflammation, chromosome segregation, translesion synthesis, or nucleolar function and dynamics (reviewed in ref. ^11^).

## Materials and methods

### Cloning, mutagenesis, cell culture, and transfections

HEK293T human embryonic kidney cells (ATCC CRL-3216^TM^) were used in different experiments. Full-length *PIDD1* (NM_145886) was amplified using reverse transcriptase PCR (RT-PCR) from cDNA generated from total RNA extracted from HEK293T cells (all reagents from Thermo Fisher scientific, Waltham, MA, USA), and cloned into mammalian expression vector pDEST47 and pDEST53, C-terminally and N-terminally GFP-tagged, respectively, using the Gateway^TM^ cloning system (Thermo Fisher Scientific, Waltham, MA, USA). In addition, an N-terminally tagged GFP construct was generated in pDEST53 with just the DD 95 amino acid residues of PIDD1 (Supplementary Fig. 1). For the N-terminally tagged GFP constructs and C-terminally tagged GFP constructs, p.Q863* and p.R815W mutations were introduced through PCR-based site-directed mutagenesis according to the manufacturer’s instructions (Quikchange Lightning site-directed mutagenesis kit; Agilent Technologies, Santa Clara, CA). Full-length wild-type (WT) CRADD (NP_001307028) was amplified using RT-PCR from cDNA generated from total RNA extracted from HEK293T cells (all reagents from Thermo Fisher Scientific, Waltham, MA, USA), and was cloned in pDsRed-Monomer-N1 vector (Clontech, Mountain View, CA). For co-localization experiments, in order to co-transfect the WT-CRADD_DsRed plus WT-PIDD1-CT-GFP, WT-CRADD_DsRed plus PIDD1-CT-GFP_Q863*, and WT-CRADD_DsRed plus PIDD1-CT-GFP R815W fusion proteins in HEK293T cells, either PolyFect (Qiagen) or polyethylenimine based transfection reagents were used (jetPEI^®^, PolyPlus, Illkirch, France) following the manufacturers’ instructions. Similarly, for co-immunoprecipitation (Co-IP) studies, the GFP-PIDD1-DD constructs and WT-CRADD_DsRed were used (see Supplementary Table 1 for primer details).

### Cell harvesting and protein extraction

One T75 flask of HEK293T cells at >90% confluence were used for all Co-IP experiments. Cells were washed with 500 μL of ice-cold phosphate buffer saline (PBS) for each flask, and harvested with a soft sterile cell scraper. Cell pellets were washed three times by resuspending the cells gently in ice-cold PBS and centrifugation at 1500 × *g* at 4 °C for 5 min. Cell pellets were stored at −80 °C until use.

For protein isolation, pellets were resuspended in lysis buffer (50 mM HEPES-KOH, 100 mM KCl, 2 mM EDTA, 0.1% NP40, 10% glycerol, 0.25 mM Na_3_VO_4_, 50 mM β-glycerolphosphate, 1 mM NaF, 1 mM DTT) and incubated on ice for 30 min. Cell debris were spun down for 15 min at 14–160,000 × *g* at 4 °C. In all, 10 U DNAse I per mL of lysate (Fermentas, Waltham, MA) were added. Protein concentrations were quantified using bicinchoninic acid protein assay kit (Pierce^TM^, Thermo Fisher Scientific, Waltham, MA). The concentration of protein in the lysate was typically ~5–10 mg/mL. An aliquot of lysate was also collected for western blot analysis and stored at −80 °C until used for further experiments.

### Co-IP and western blotting (WB)

Lysate of co-transfected HEK293T cells as prepared above were used for all Co-IP experiments. C-terminally/N-terminally GFP-bound PIDD1 WT, Q863*, and R815W were immuoprecipitated (IPed) using monovalent matrix agarose Nanobeads GFP-Trap^®^ (ChromoTek GmbH, Martinsried, Germany) following the manufacturer’s instruction. The IPed proteins were ran on 4–20% sodium dodecyl sulfate polyacrylamide gel electrophoresis (SDS-PAGE), followed by western blot using (1) anti-GFP antibody (Thermo Fisher Scientific, Waltham, MA) in order to analysis PIDD1 auto-cleavage in PIDD1-N and molecular size variation between WT and Q863* protein (Supplementary Fig. 1A); (2) anti-GFP antibody (Thermo Fisher Scientific, Waltham, MA) and Living Colors® DsRed Polyclonal Antibody (Clontech, Mountain View, CA.) to identify Co-IP-ed CRADD and PIDD1-WT, PIDD1_Q863*, and PIDD1_R815W (Supplementary Fig. 1B, C) from co-transfected HEK293T cells.

### Cell-based protein degradation

In order to check the stability of PIDD1 protein carrying the identified missense, nonsense, and splice mutations, in comparison to WT PIDD1, we transfected HEK293T cells as described previously, and after 24 h added 10 µg/mL cycloheximide (CHX; Sigma-Aldrich, St Louis, MO) to prevent translation of new protein, plus or minus proteosomal inhibitor MG-132, and harvested at the indicated times. Samples collected at each time point were then analyzed by western blot, using anti-FLAG-M2 antibody (Sigma-Aldrich, St Louis, MO).

### Fluorescence confocal microscopy

All co-transfected cells were fixed after 48 h using 4% paraformaldehyde followed by 2× washes with PBS, and then counter-stained with the AT-selective DNA dye DAPI (4′-6′-diamidino-2-phenylindol). *Z*-stack images acquired with a frame size of 512 × 512 pixels were used to study PIDD1-CRADD co-localization. Confocal microscope, Olympus FV1200 using ×63/1.3 NA oil objective were used to capture DAPI (heterochromatin) at 405 nm, 488 nm for GFP (recombinant PIDD1-GFP fusion protein), and 588 nm for DsRed (recombinant CRADD-DsRed fusion protein).

### Data analysis

Bio-Rad’s Quantity One^®^ ID analysis software and Image Lab^TM^ software were used to process gel images. For cellular images Olympus FluoView software to calculate Pearson’s correlation coefficient (PCC) for the co-localization of chromocenters (DAPI) and recombinant protein (GFP & DsRed monomer N1) was used. The descriptive statistical data analysis was calculated using Microsoft Excel and two-tailed Student’s *t-*test for statistical significance was calculated using GraphPad online tool (GraphPad Software Inc., San Diego, CA).

### Exon-trap assay for c.2275-1G>A mutation

Exon 15 of the wild-type *PIDD1* gene, as well as portions of its flanking regions (235 bp upstream and 368 bp downstream), were PCR-amplified from the genomic DNA of an unrelated (and unaffected) individual, restriction digested with *Sal*I and ligated into the MCS of the pET01 vector (MoBiTec GmbH). The pET01 MCS is located between two bordering exons. In order to generate the pET01-PIDD1 vector with the non-synonymous mutation in intron 11 (c.2275-1G>A), site-directed mutagenesis was performed. Briefly, PCR using mutagenic primers was used to induce the base substitution, followed by digestion of the parental template using *Dpn*I, phosphorylation of the mutagenic amplicon by T4 PNK (NEB), and circularization using Quick T4 DNA Ligase (NEB, Ipswich, MA). Following verification by Sanger sequencing, the wild type, mutant, and control (empty pET01) vectors were separately transfected (Lipofectamine 3000 (Thermo Fisher Scientific, Waltham, MA)) into HEK293T cells. Twenty-four hours post-transfection, RNA was extracted and, after first-strand cDNA synthesis with random hexamers, RT-PCR was performed using primers complementary to the pET01 exons that border the MCS. Following gel electrophoresis, bands were excised, purified, and submitted for Sanger sequencing. All primers are listed in Supplementary Table 2.

### PIDD1 overexpression and functional analysis

C-terminally FLAG-tagged versions of either WT *PIDD1* or *PIDD1* point mutants Q863*, R815W, c.2275-1G>A (splice acceptor), or G876S, a variant found in heterozygous form in an unrelated ID patient (John B. Vincent, unpublished data) were generated by site-directed mutagenesis and cloned into pcDNA5 expression plasmid by PCR cloning. The c.2275-1G>A mutant variant missing exon 15 was generated by producing a PCR fragment using a CMV promoter-specific forward primer and a selective hPIDD1_*BamH*I_Exon_DD reverse primer (see Supplementary Table 3) with an additional C-Terminal *BamH*I restriction site to clone the PCR fragment back into the original pcDNA5 plasmid, after *BamH*I digestions, thereby keeping the C-terminal FLAG-tag intact. To allow reconstitution of PIDD1 KO cells^10^ silent mutations were additionally introduced in the PAM sequence to neutralize the originally used sgRNA (ccccatcaccatccgctatcggc) in these cells (FAVA REF G&D).

The functionality was assessed by transiently transfecting the constructs into the PIDD1 KO U2OS or A549 cell lines^10^ in duplicates, using Metafectene (Biontex Laboratories GmbH, Munich, Germany) as described in the user manual (six-well plate, 400,000 cells/well). After overnight transfection, one subset of the duplicate was treated with 2 μM ZM447439 for 48 h, while the other was left untreated. Subsequently cells were lysed and immunoblotted for MDM2 (MAY-113; Invitrogen, Thermo Fisher Scientific, Waltham, MA) and GAPDH (CS14C10; Cell Signaling Technology, Danvers, MA). Construct expression and processing was detected in HEK293T cells using Sigma anti-FLAG-M2 antibody. For immunoprecipitation, FLAG-M2 agarose beads (Sigma) were used to enrich FLAG-tagged PIDD1 variants.

### Western blotting

Cell lysates were prepared using lysis buffer containing 50 mM Tris pH 8.0, 150 mM NaCl, 0.5% NP40, 50 mM NaF, 1 mM Na_3_VO_4_, 1 mM PMSF, one tablet protease inhibitors (EDTA free; Roche) per 10 mL and 30 μg/mL DNaseI (Sigma-Aldrich, St. Louis, MO). Protein concentration was measured by Bradford analysis (500-0006, Bio-Rad Laboratories, Hercules, CA). SDS-PAGE was performed using a 12% polyacrylamide gel prepared with the Bio-Rad Mini-Protean system. Subsequent electro-blotting was conducted using AmershamTM HybondTM-ECL nitrocellulose membranes (GE Healthcare, Chicago, IL). Membranes were then blocked in 5% milk powder PBST and immunoblotted for MDM2 (Invitrogen MAY-113), CRADD (4B12, LSBio), or GAPDH (Cell Signaling CS14C10). Construct expression and processing was detected in HEK293T cells using Sigma anti-FLAG antibody.

### Animal experiments

Mice were generated as previously described (*Pidd1*^*tm1.Anvi*^)^18^, with exons 3–15 removed. KO mice and WT littermates underwent a physical exam at the age of 3 and 12 months. For the 3-month-old group, 10 male WT and 9 male KO mice were studied. For the 12-month-old group, 8 female WT mice and 8 female KO mice were studied. This exam included observation of ambulation in a novel cage, hearing (response to finger snap) and visual ability (visual cliff), condition of fur and whiskers, body weight, motor skills (beam walk), and muscular strength (wire hang test). Ambulation was observed for 5 min, looking for overall activity, stereotypies, seizures, and other abnormal behavior. The beam walk test was performed on square wooden sticks with 1 and 2 cm diameter and 1 m length. Mice were placed in the center, and the time needed to reach the end was measured (three repetitions, best taken). The wire hang test was performed by placing mice on a wire grid, which was inverted after the mouse grabbed hold. Maximum duration was 60 s. For younger (3 months) mice the open field, light-dark, and elevated plus maze tests were also performed. Mice were transferred to the ante-room of the testing facility 24 h before the commencement of experiments. The free access to food and water, the climate, and the light–dark cycle were kept constant. The tests were performed between 9 a.m. and 1 p.m. All tests were video monitored and evaluated by an experimenter blinded to the genotype of the animals. The procedures for the open field, elevated plus maze, and light–dark choice tests were performed as published earlier^22^ in accordance with the recommendations of EMPRESS (European Mouse Phenotyping Resource of Standardised Screens; http://empress.har.mrc.ac.uk).

In short, the open field arenas had a size of 50 × 50 cm and were illuminated to 150 lux. Mice were observed over 10 min, measuring time, distance traveled, and number of entries into three subfields: center (central 16% of overall area), border (8 cm along walls), and intermediate.

The light–dark test was performed in the same arenas with a black box inserted, which covered 1/3 of the area. Light was set to 400 lux and mice tested for 5 min measuring time, distance traveled, and number of entries into the light compartment.

The elevated plus maze test consisted of four arms, two closed (20 cm walls) and two open arms, each 50 × 5 cm in size elevated about 80 cm above ground. Exploratory activity on the open arms was tested over 5 min at 180 lux.

### Bioinformatic transcriptomic analyses

In order to better understand the possible connection between *PIDD1* and the brain developmental phenotypes observed in individuals with mutations disrupting PIDD1 function, we used publically available transcriptomic datasets (gene expression microarray and RNAseq) for *PIDD1*, and co-expression with related genes to explore commonalities in temporaspatial expression. Firstly, we used data from the Genotype-Tissue Expression (GTEx) project to explore gross anatomical gene expression for humans through the GTEx Portal (https://www.gtexportal.org/home/). Secondly, we used Allen Brain Atlas data (www.brainspan.org) to look at temporospatial mRNA expression for human brain from 8 weeks post-conception to 40 years of age, using RNAseq and gene expression microarray^23^, and from RNAseq data from the PsychEncode dataset^24^. Adult Human transcriptomic comparisons were performed with the Allen Human Brain Atlas data^25^. Co-expression analysis of BrainSpan datasets was performed as described previously^26^ (https://hbaset.msl.ubc.ca/). Thirdly, we used single-cell RNAseq data from fetal and postnatal mouse brains, for which cell types have been classified according to spatial and taxonomical cluster analysis (www.mousebrain.org)^27,28^. Data were analyzed from the single-gene perspective (*PIDD1*/*Pidd1* alone), also with PIDDosome interactors (*PIDD1*/*Pidd1*+*CRADD*/*Cradd*+*CASP2*/*Casp2)*, or with a gene set of PIDD1 interactors (P gene set: *PIDD1*, *CRADD*, *CASP2*, *MADD*, *FADD*) plus genes relevant to lissencephaly (L gene set: *RELN*, *TUBA1A*, *NDE1*, *KATNB1*, *CDK5*, *ARX*, *DCX*, *LPHN1*, *LPHN2*, *LPHN3*).

## Results

### Familial history and clinical observations for *PIDD1* mutation families

Figure 1A shows pedigree information and segregation pattern of five consanguineous families with mutations in *PIDD1*. Family M278, with two affected siblings, has a splice acceptor mutation (Chr11:800015C>T; NM_145886.3:c.2275-1G>A), just ahead of the penultimate (14th) exon. The variant is not present in the gnomAD database. Family M8700004, with three affected siblings, has a missense mutation (Chr11:799846G>A; NM_145886.3:c.2443C>T; p.Arg815Trp), converting a residue situated within the DD that is positively charged across eutherian mammalian evolution to a non-polar residue (Supplementary Fig. 2). This variant (rs758859772) is present in the gnomAD database, with an allele frequency of <0.0001, with no homozygous occurrences. Indian family Manipal-1 has not been reported previously. The female proband presents with mild ID, and has the homozygous nonsense mutation (Chr11:800770>A; NM_145886.3: c.1909C>T; p.Arg637*; see Supplementary Fig. 3 for electropherograms)). This variant (rs578222814) is present in the gnomAD database, with an allele frequency of <0.0001, with no homozygous occurrences. None of these variants are present in the GMEV.

A more detailed clinical analysis of the five families is provided in the Supplementary Information and the genomic location on Chr11 as well as intron–exon structure of the *PIDD1* gene are depicted in Fig. 1B. Briefly, eight out of eleven affected family members (for whom clinical information was available) were reported to have epilepsy (generalized tonic–clonic seizures). Clinical magnetic resonance imaging (MRI) reports were available for two affected family members with the Gln863* mutation, as well as the proband from Manipal-1 with Arg637*. Although both reports from AS110 indicated normal anatomy, our own re-examination of the images indicated: in AS110 IV-3, a 30-year-old male, subtle lissencephaly of the cortical layers of the cerebral hemispheres, most pronounced in bilateral frontal lobes (Fig. 1C–E); in AS105 III-3, a 5-year-old male, mild decreased volume of the body and splenium of the corpus callosum. MRI from Manipal-1 II-3 shows broad smooth gyri in bilateral fronto-temporal lobes, with reduced sulcations, suggestive of the lissencephaly–pachygyria complex. Thus, as with *CRADD*, mutations in *PIDD1* may also be associated with lissencephaly. Although normal gait was reported for all available affected members, bradykinesia was evident in AS110 IV-1 and IV-3, and slurred speech was reported for family M8700004. Affected individuals were classified as moderate to severe intellectually disabled. Vision and hearing were reported as normal, although one individual was reported as having convergent strabismus. Aggressive behavior was a common feature in the Gln863* families, but not the splice site family. Psychosis was also reported for the affected Arg815Trp family members by the mother, and confirmed after evaluation by a psychiatrist.

### Mutations in the PIDD1 DD cause mis-localization of CRADD

As with the nuclear pore protein Nup98/96, PIDD1 protein auto-processes itself by an intein-like mechanism, which gives rise to a leucine-rich PIDD1-N and PIDD1-C fragment containing the DD. PIDD1-C is further auto-cleaved into PIDD1-CC^29^. Figure 2A highlights the autoprocessing sites and residue count of the resulting cleaved PIDD1 proteins. All four mutations (plus the G876S variant) are either within or ahead of the DD (Q863*, R815W, G876S, R637*), or impacting the DD (c.2275-1G>A) by potentially impairing splicing, leading to a premature stop and protein truncation (Fig. 2B).

**Fig. 3:**
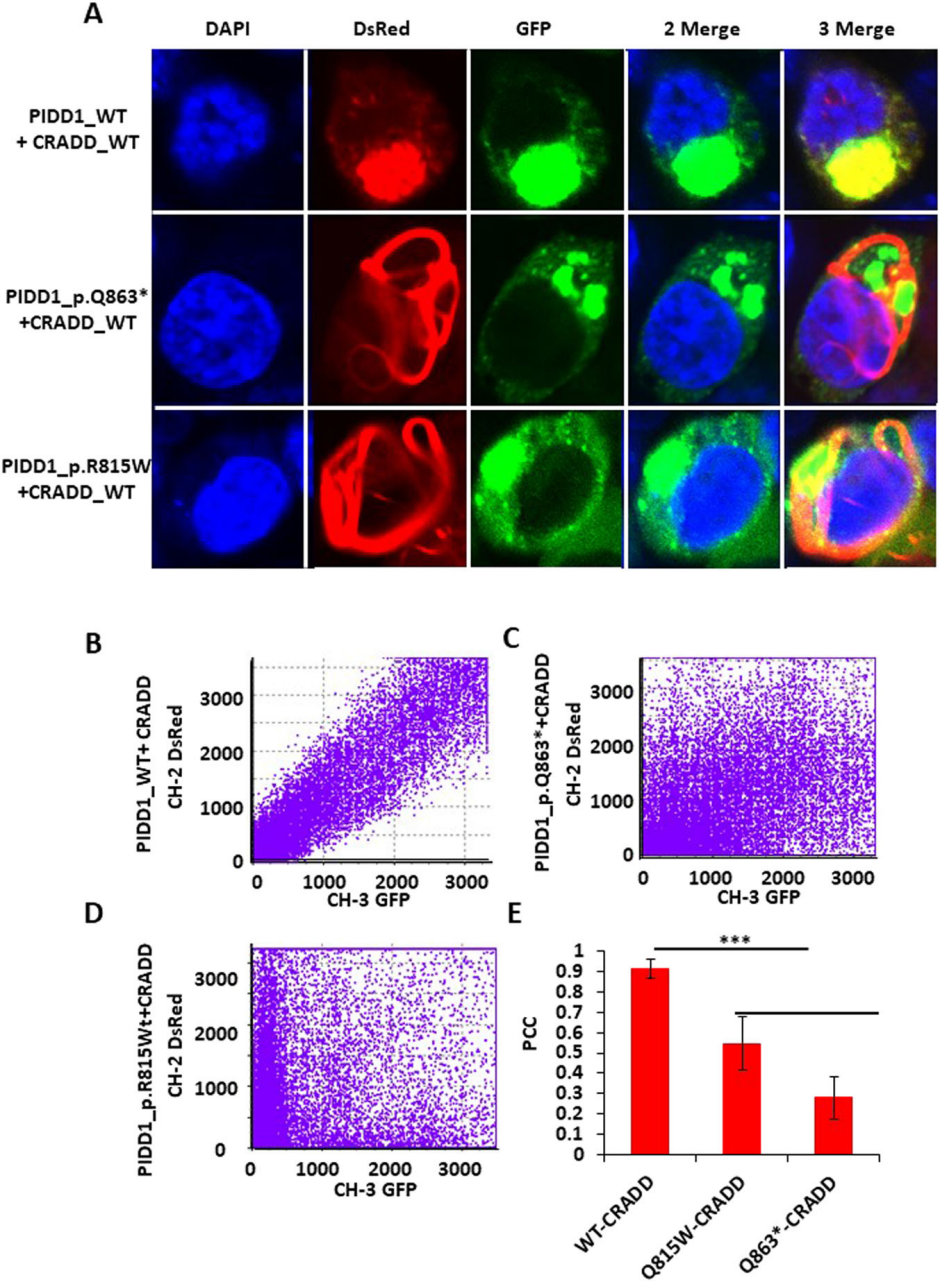
Co-localization analysis in HEK293T cells co-expressing PIDD1-FL C-terminally fused with GFP and CRADD C-terminally fused with DsRed protein. **A** Column 1 DAPI, column 2 DsRed, column 3 GFP, column 4 two merge (DAPI-GFP), and column 5 three merge (DAPI-GFP-DS-Red). (Top row) 512 × 512 image stacks showing full-length WT PIDD1-GFP fusion protein and full-length WT CRADD-DsRed fusion protein. (Middle row) 512 × 512 image stacks showing full-length Q863*PIDD1-GFP fusion protein and full-length WT CRADD-DsRed fusion protein. (Bottom row) 512 × 512 image stacks showing full-length R815W PIDD1-GFP fusion protein and full-length WT CRADD-DsRed fusion protein. **B**–**D** Plot representing overlapping pixels of *X*-axis (CH2-DS-red) and *Y*-axis (CH3-GFP) of WT-CRADD and PIDD1-WT, PIDD1-R815W, and PIDD1-Q863*, respectively. **E** Quantitative PCC values of same as **B**–**D**. *n* = 10 ±SEM bar shown ***p ≤ 0.0001; unpaired Student’s *t*-test.

PIDD1–CRADD interaction is crucial for PIDDosome assembly^7,30,31^. To analyze the cellular localization of WT and mutant PIDD1 as well as their ability to interact with CRADD, we conducted co-localization experiments by overexpressing wild-type CRADD with a C-terminal DsRed tag along with PIDD1-WT, PIDD1-Q863*, and PIDD1-R815W with C-terminal GFP tag (Fig. 3). Overexpressed WT and mutant PIDD1 localize in the cytoplasm but mutant PIDD1 protein, and PIDD1-p.Q863* in particular, appeared to be more scattered, whereas WT PIDD1 appears mainly within a single cluster (Fig. 3A, column 3). Interestingly, the localization pattern of overexpressed CRADD-DsRed fusion protein was also markedly different and distinct in the cells overexpressing mutant PIDD1 (Fig. 3A, column 2). Merged images of GFP and DsRed clearly show mis-localization of CRADD and mutant PIDD1 proteins (Fig. 3A). Scatter plots of fluorescence intensity of GFP and DsRed channels represents a clear pixel-by-pixel co-localization pattern of both proteins for WT PIDD1 (Fig. 3B), but in the case of mutant protein no such co-localization pattern was observed (Fig. 3C, D). Quantitatively, a highly positive correlation was observed between overexpressed WT-CRADD and WT-PIDD1, and low to negligible correlation for WT-CRADD and PIDD1-R815W and PIDD1-Q863* (Fig. 3E). PCC values of CRADD-PIDD1-WT, CRADD-PIDD1_R815W, and CRADD-PIDD1_Q863* were 0.913 ± 0.048, 0.547 ± 0.131, and 0.278 ± 0.104, respectively (averaged *n* = 10; *p* value ≤0.0001; unpaired Student’s *t*-test (Fig. 3E).

### DD mutations do not prevent auto-proteolysis or impact stability of PIDD1

First, we explored the relevance of the splice acceptor site mutant. Since access to fresh RNA from the Iranian family M278 was not feasible, and thus we were unable to check directly the effects on splicing of the c.2275-1G>A mutation and any resulting changes to the *PIDD1* open reading frame, we opted to construct an exon-trap construct as a model system to investigate the likely effects of the mutation and confirm in silico predictions of exclusion of exon 15 from the mRNA. Exon-trap analysis showed skipping of exon 15, with direct splicing from exon 14 to the terminal exon 16 (Supplementary Fig. 4) resulting in the substitution Arg759Gly with premature termination at codon 760 (p.Arg759Glyfs*1) leading to the exclusion of the entire DD (Fig. 2B).

**Fig. 4:**
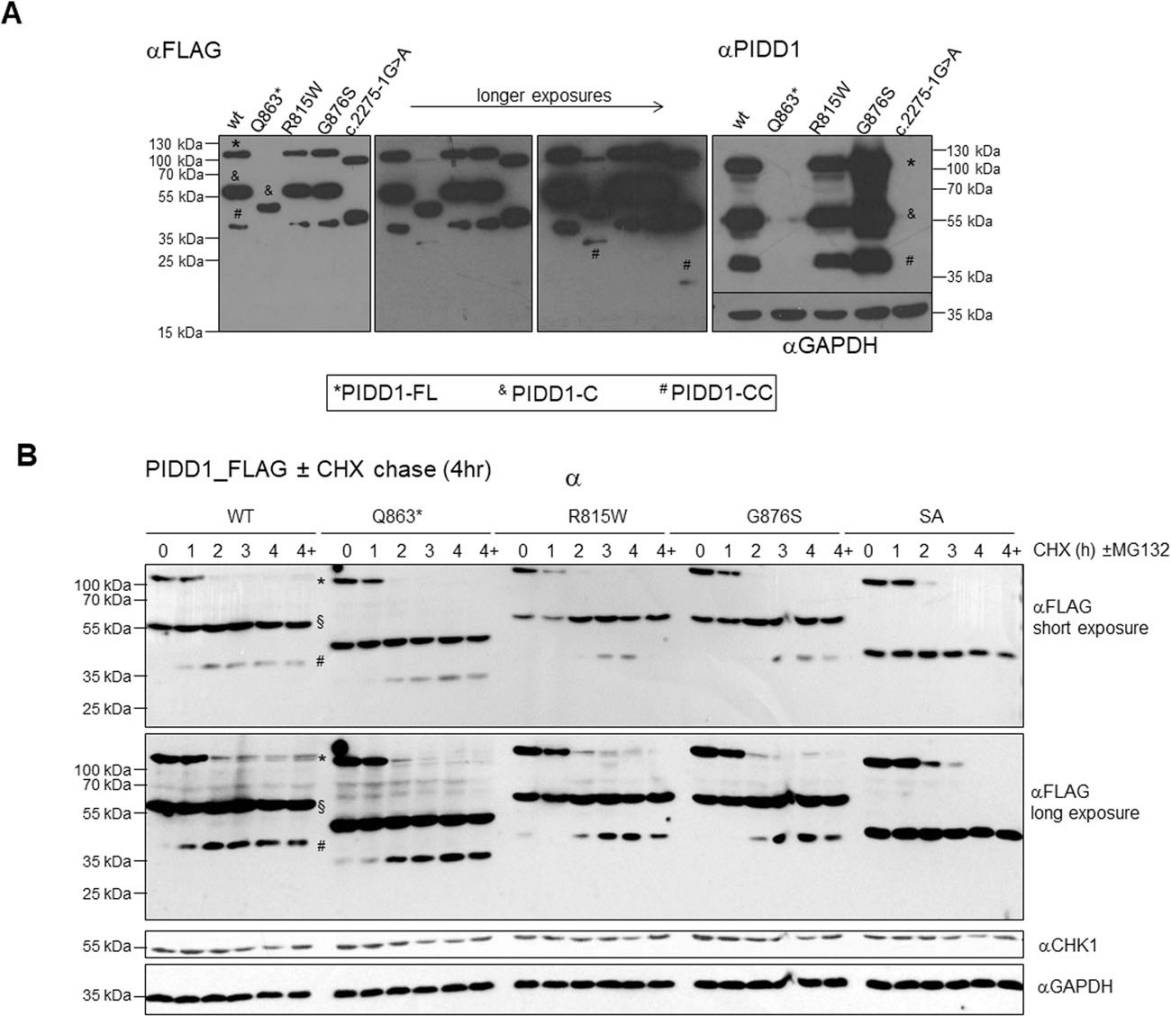
PIDD1 mutations, autoprocessing and stability. **A** PIDD1 mutations do not disrupt autoprocessing. HEK293T cells were transiently transfected with the indicated FLAG-tagged variants of PIDD1 and analyzed by western blotting using either anti-FLAG-M2 or an anti-PIDD1-specific antibody. **B** Cell-based protein stability of mutant versus wild-type PIDD1. HEK293T cells were transiently transfected with vectors encoding FLAG-tagged PIDD1 wild-type (WT), and DD mutants Q863*, R815W, G876S, or the splice acceptor (SA) mutant. After 24 h the cells were treated with cycloheximide (CHX), alone or in combination with the proteasomal inhibitor MG-132, for the times indicated, and were then processed for western blotting using and anti-FLAG antibody, in a 4-h chase experiment. A longer, 12 h chase experiment was also performed, and results shown in Fig. S1. Reprobing with an antibody-recognizing GAPDH, or CHK1, was done to confirm comparable protein loading. PIDD1-FL (*), -C (§), and –CC (#) are indicated.

Next, we transiently transfected HEK293T cells with C-terminal FLAG-tagged PIDD1 in its WT or various mutant forms. This analysis revealed normal processing of PIDD1 in case of the R815W or G876S variant, where PIDD1-FL, -C and -CC were present at similar levels. However, PIDD-Q863* appears to be poorly expressed and showed little PIDD1-FL or PIDD1-CC species, both only visible under extended exposure (Fig. 4A). The splice acceptor mutation (c.2275-1G>A), however, appeared to affect mainly the formation of PIDD1-CC. As a note on the side, probing the same lysates with the PIDD1-specific antibody clone ANTO1 suggests that the epitope recognized must lie within the DD downstream of Q863 (Fig. 2B). Together we conclude that single point mutations G876S and R815W do not affect autoprocessing or protein stability, while mutations that truncate the DD can interfere with protein stability (Q863*) or interfere with autoprocessing and efficient generation of PIDD1-CC, as in case of c.2275-1G>A, that leads to exon skipping and premature stop in position 760.

Cycloheximide-chase assays were performed to check whether the PIDD1 mutant proteins undergo accelerated protein degradation; however, it appears that WT and mutant PIDD1 are relatively stable, and, even when tracked over 4 h (Fig. 4B) or 24 h (Fig. S1 (D), do not show substantial degradation, and any differences between WT and mutants are minimal. Thus, the PIDD1 mutations identified do not significantly impact either autoprocessing or protein stability.

### Pathologic PIDD1 mutants fail to interact with CRADD and activate caspase-2

**Fig. 5:**
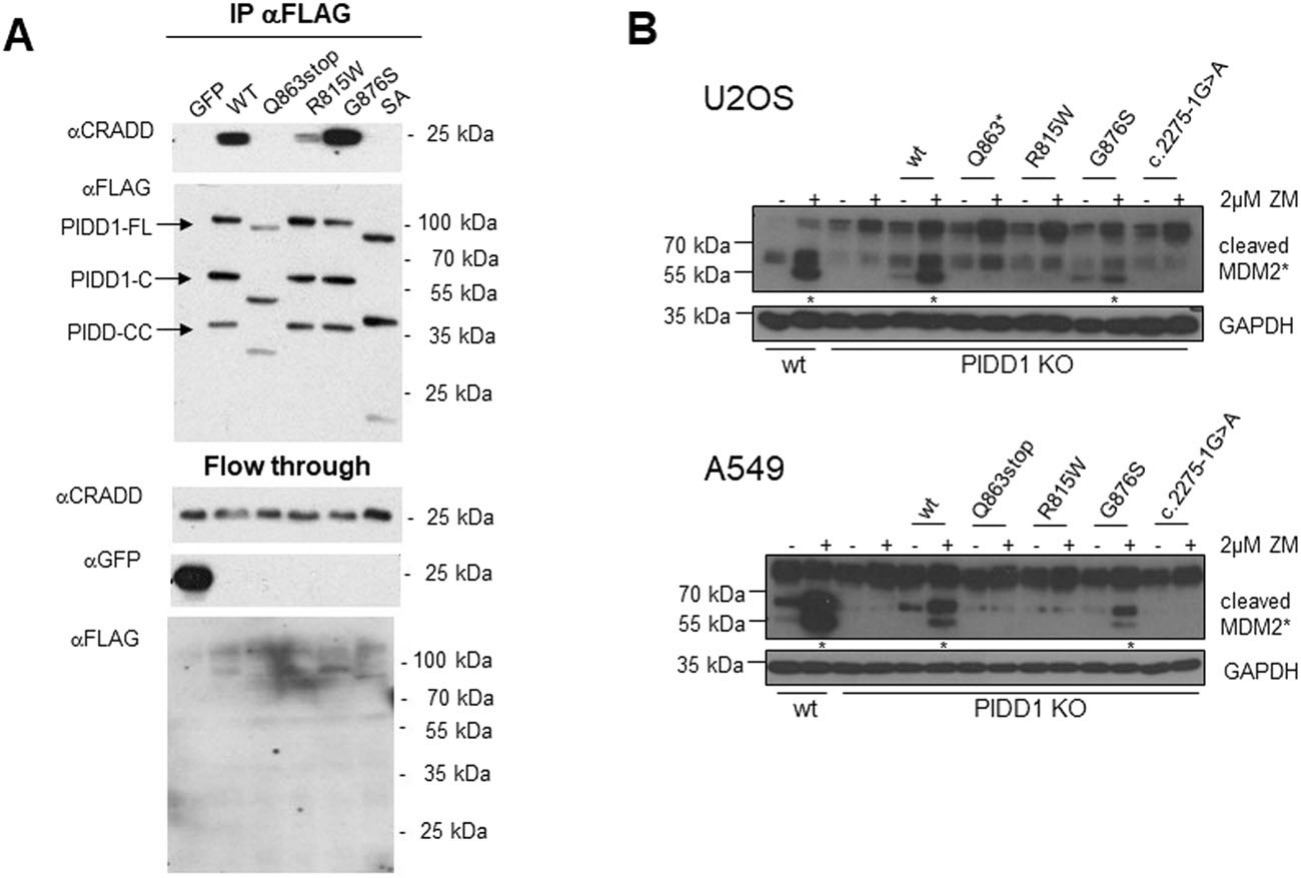
The effect of PIDD1 mutations on interacton with CRADD and caspase-2 activation. **A** PIDD1 immunoprecipitation pull down of CRADD. HEK293T cells transfected with vectors encoding GFP or FLAG-tagged WT or mutant PIDD1 were lysed after 24 h, and subjected to immunoprecipitation, followed by western analysis using antibodies recognizing the FLAG-tagged PIDD1, or CRADD, in the IP sample or flow through. **B** Functional effect of PIDD1 mutation on Caspase-2 activity through MDM2 cleavage assay. PIDD1-deficient U2OS or A549 cells generated by CRISPR/Cas9 technology were transiently transfected with the same constructs used in **A** and treated with the AuroraB kinase inhibitor ZM447439 to induce cytokinesis failure and PIDDosome activation. Cell lysates were probed with an antibody recognizing the cleaved variant of MDM2. Reprobing with an antibody recognizing GAPDH was done to confirm comparable protein loading.

It was previously shown that PIDD1 activates caspase-2 in the PIDDosome in a CRADD-dependent manner in response to cytokinesis failure or accumulation of extra centrosomes^10^. In turn, caspase-2 cleaves MDM2 which leads to p53 activation and p21 induction. Hence, we tested if PIDD1 mutants can still interact with CRADD. To this end, FLAG-tagged versions of PIDD1 were overexpressed in 293T cells followed by immunoprecipitation analysis. This analysis revealed that exogenous PIDD1 can interact with endogenous CRADD in its WT or G876S mutant form, while the premature stop or splice site-induced truncation mutants lost interaction with CRADD and the R815W point mutant showed strongly reduced binding (Fig. 5A). Consistent with this WB of N-terminally GFP-tagged PIDD1-Q863* showed a faster migrating PIDD1-FL band compared to WT, but similar size to WT for a stronger band at ~75 kDa, likely representing the PIDD1-N cleavage product (Supplementary Fig. 2A). The Co-IP of GFP-tagged PIDD1-DD with CRADD demonstrated ablation of interaction for the Q863* and R815W mutations (Supplementary Fig. 2B, C).

To explore the potential consequences of mutation for PIDDosome function we performed reconstitution assays where PIDD1-deficient U2OS osteosarcoma and A549 lung-adenocarcinoma cells were used to transiently express sgRNA resistant WT, mutant, or truncated PIDD1 proteins. Cleavage of MDM2 by active caspase-2 was used as a functional readout in western analyses. Using this assay, we can conclude that all PIDD1 mutants bar the G876S variant have lost the ability to activate caspase-2, and hence constitute loss-of-function alleles. The G876S mutant appears to act as a mild hypomorph in this assay (Fig. 5B).

### Mouse phenotyping

**Fig. 6:**
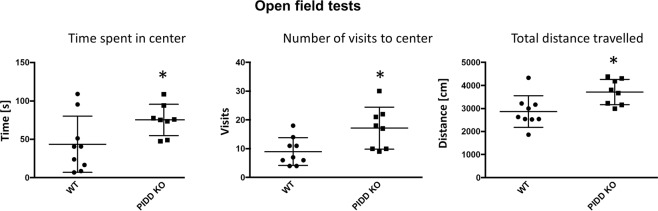
Open field testing on *Pidd1* knockout mice. Open field testing on *Pidd1* KO mice (*n* = 9) and WT littermates (*n* = 10), as performed on 3-month-old male mice, measuring the time spent in the center of the field, the number of visits to the center of the field, and the total distance traveled by the mice during the testing perion. The Mann–Whitney *U* test was performed, and the asterisk indicates statistical significance (*p* < 0.05). Other tests performed for which results did not reach statistical significance are provided in Supplementary Fig. 5.

The physical examination of the *Pidd1*-deficient mice did not reveal any differences between KO and WT genotypes, neither at 3 nor at 12 months of age. Overall, KO mice tended to start ambulation of the novel cage slower than WTs, but did not show abnormal behavior or signs of distress or panic. Fur and whiskers were generally well, body weight was similar (25 ± 1.6 vs. 27 ± 1.3 at 3 months and 31 ± 4.1 vs. 29 ± 3.5 at 12 months for WT and KO, respectively. Data represent mean ± SD). Vision was intact at 3 months of age, but impaired at 12 months of age. However, this is normal for the C57BL/6N strain. At 3 months of age, four out of nine KO mice did not stay the entire time in the wire hang test, while all WT mice did. However, the difference did not reach statistical significance. At 12 months of age, one out of eight KO and two out of eight WT mice dropped before 60 s. The light–dark and elevated plus maze tests did not reveal any difference (Supplementary Fig. 5). By contrast, a mild but statistically significant anxiolytic-like effect was observed in the open field test (see Fig. 6).

### Bioinformatic transcriptomic analyses

Gross anatomical transcriptional surveying through the GTEx data shows broad expression of human *PIDD1* mRNA in multiple tissues, but with higher expression in brain (cerebellum) (Supplementary Fig. 6). BrainSpan exon microarray and RNAseq data analysis and PsychEncode RNAseq data analysis for *PIDD1* alone, for PIDDosome genes (*PIDD1*+*CRADD*+*CASP2*), or for PIDDosome and lissencephaly related (P+L) genes, shows higher expression and co-expression during early human development (see heat maps in Supplementary Fig. 7). Co-expression of *PIDD1*+*CRADD*+*CASP2* in six adult brains (through area under the curve (AUC) analysis) is mainly restricted to corpus callosum, cerebellum, and thalamus (Supplementary Fig. 8).

Analysis of the single-cell RNAseq data from developing mouse brains^27,28^ shows overall sparse neuronal expression for *Pidd1*, but with a small cluster in *Slc17a6*-co-expressing (*Slc17a6* is used as a marker for glutamatergic neurons) forebrain neurons at age E12−16 post-conception days (pcd), but with higher density transcription among cells classified as radial glia, neural crest, choroid, and ectodermal (see Supplementary Fig. 9a Mouse brain cells reference map, and 9b). This is in clear contrast to other PIDDosome components *Cradd*, and *Casp2*, which are more abundantly and ubiquitously expressed in neuronal and non-neuronal cells (Supplementary Fig. 9c, d). Co-expression analysis of *Pidd1* + *Cradd* + *Casp2* is highly specific. Ranking co-expression of the three genes by area under the curve (AUC) analysis for postnatal mouse^27^ (but data not available for fetal mouse) shows most of the co-expression of *Pidd1*, *Cradd*, and *Casp2* being in sympathetic and peripheral neurons and glia (e.g. in cholinergic sympathetic neurons (SYCHO1), and cholinergic enteric neurons (ENT8) and glia (ENTG1,3,5)). Granule neuroblasts from dentate gyrus is the only cell cluster from the central nervous system (CNS) with significant co-expression (*p* < 0.05) (Supplementary Table 4).

It should be noted that gene transcription profiles do not necessarily correlate directly with expression of the encoded protein; however, such data are frequently used as a proxy for protein expression data, given the challenges of obtaining such high-resolution information for proteins.

## Discussion

Mutations in the *CRADD* gene have been linked with lissencephaly, a condition in which defective neuronal migration leads to cortical underdevelopment and reduced or absent gyri, resulting in ID and/or other neurodevelopmental disorders^2,3^. Examination of MRI from two affected individuals with the *PIDD1* Gln863* mutation did show subtle signs of lissencephaly in one (the elder) of the two individuals; however, the images available to us were poor quality. Brain MRI from the Manipal-1 proband with the Arg637* mutation also indicated lissencephaly, more specifically pachygyria, as well as shortened/thickened corpus callosum. This adds weight to lissencephaly being a common feature for both CRADD and PIDD1-related disorders. Both CRADD and PIDD1 proteins form part of the complex known as the PIDDosome—a regulator of cell death in response to DNA damage^7^. Of note, PIDD1 and CRADD have been reported to activate caspase-2 in various cellular contexts, including DNA damage or centrosome amplification in cancer cells as well as amyloid-induced neuronal toxicity^8,11^. Remarkably, constitutive post-translational self-processing of PIDD1 gives rise to PIDD1-N (~48 kDa) and PIDD1-C (~51 kDa), which is further processed into PIDD1-CC (~37 kDa), needed for caspase-2 activation within the PIDDosome^29^. PIDD1-N contains a leucine-rich domain while PIDD1-C contains a DD from residues 774–893. Serine residues at S446 (generating PIDD1-C) and S588 (generating PIDD1-CC) are involved in this autoprocessing mechanism^29^. In our study we have shown that none of the genetic alterations reported here prevent self-processing of PIDD1, but differences in stability become apparent in PIDD1-CC variants with a truncated DD. This suggests that the effect of the *PIDD1* mutations that have been associated with ID^19,21^ are most likely due to the loss of function of PIDD1-CC, as PIDD1-C can be still be generated at rates comparable to WT (Fig. 4). Caspase-2 recruitment and activation in the PIDDosome is critically dependent on PIDD1–CRADD interaction, as a complete loss of caspase-2 activation was observed in the several CRADD and PIDD1 mutants^32^. Here, either directly using Co-IP or indirectly using MDM2 cleavage as the readout, we confirm the loss of PIDD1–CRADD interaction due to *PIDD1* DD mutations R815W and Q863*, while the G876S variant (found only in heterozygous form in a single unrelated affected individual) has no significant impact. Despite showing relatively poor amino acid residue conservation across vertebrate families at R815 (see Supplementary Fig. 3), the R815W DD substitution shows this effect to a similar degree compared with the Q863*. Consistently, these mutants were no longer able to process MDM2 effectively. Currently, we have not documented that mutant PIDD1-C is also functionally impaired. However, as NF-κB activation by PIDD1-C also depends on DD–DD interaction within RIP1 and mutant PIDD1 fails to interact with CRADD, it is fair to speculate that this function of PIDD1-C would also be compromised to some degree.

Degradation assays using cycloheximide suggest that there is negligible effect of the PIDD1 mutations on protein stability or longevity. This supports our conclusions that the effect of these mutations is through loss of DD function. However, it is quite possible that other mutations in PIDD1 will impact the protein stability, as has been observed for the CRADD Gly128Arg mutation^2^.

Although the phenotypic consequences of *Pidd1* knockout in mice did not recapitulate the cognitive deficit seen in the patients with biallelic disruption of the *PIDD1* C-terminal DD, there were mild but significant anxiety phenotypes noted. A recent meta genome-wide association study across several anxiety-related neurodevelopmental disorders (autism spectrum disorder, attention deficit/hyperactivity disorder (ADHD), obsessive compulsive disorder, and Tourette Syndrome) including over 93,000 study participants showed genome-wide significant association at *PIDD1*^33^. Further, it has been reported that a risk allele for ADHD at *PIDD1* (rs7104929, allele G) correlates with significantly increased methylation at promoter and enhancer epigenetic marks and decreased transcription of the gene^34^. Recent whole-genome sequencing efforts for autism spectrum disorder proband/mother/father trio through the Autism Speaks/MSSNG study also has some evidence for *PIDD1* as a rare risk gene—out of ~10,000 trios sequenced, a single affected individual (AU2798301) is reported to show compound heterozygous variants: maternal allele: chr11:802296G>C; NM_145887:exon6:c.C1075G; p.(Arg359Gly); paternal allele: chr11:802684G>A, NM_145887:exon4:c.C917T:p.Pro306Leu. The respective allele frequencies in the gnomAD control database are 8E−6 and 4E−6, with no homozygotes observed. Predictions for these missense variants are given as “high” and “medium” damaging, respectively (https://research.mss.ng). It is also worth noting that copy number variants across *PIDD1* reported in the DECIPHER database (compiled in ref. ^34^) include 10 duplications and 1 triplication, 5 of which are de novo, in individuals with ID, global developmental delay, autism, delayed speech and language development, and macrocephaly with postnatal growth retardation. Thus, there is accumulating evidence of a role for *PIDD1* in neurodevelopmental disorders spanning multiple diagnoses, and with a variety of inheritance patterns.

It is worth noting that, for *Cradd*−/− mice, Di Donato et al.^2^ reported no significant CNS phenotype, and megaloencephaly but no cortical lamination defect, contrasting with the lissencephaly and ID reported in *CRADD* mutation patients. Brain anatomical evidence for the human *PIDD1* mutations comes from both *PIDD1* families for which MRI brain images were available, and *Pidd1*−/− mouse brain anatomical analysis has not been performed yet, but it is also clear that, as with *CRADD*/*cradd*, the ablation of mouse *Pidd1* does not appear to cause the degree of cognitive deficit seen in humans with disruption of the C-terminal PIDD1 DD and PIDD-CC function. It should be noted that the fetal mouse brain cell-type RNAseq analyses suggest that *Pidd1* expression in neurons is very restricted, and the degree of transcriptional overlap between *Pidd1*, *Cradd*, and *Caspase-2* (*Casp2*) in CNS cells is very low. This could explain the lack of CNS-related phenotypes for the *Cradd* and *Pidd1* mice, but could also limit any study of *PIDD1* mutations in neuronal cells. We posit that there may be higher overlapping expression of these genes in developing human CNS, which may be relevant to the etiopathology of *PIDD1* mutations. The anatomical RNAseq and microarray data from the human BrainSpan study^23^ and PsychEncode RNAseq study^24^ certainly suggest that co-expression of *PIDD1*, *CRADD*, and *CASP2* is high in many brain regions in the developing human brain (see Supplementary Fig. 7). However, as with mice^28^, expression of *PIDD1* in specific cell types in human fetal cerebrum and cerebellum is low and very sparse, but co-expression data and data for other brain regions are not yet available^35^.

Whether the etiological pathway for *PIDD1* and *CRADD* mutations in neurodevelopmental disorders is united through the PIDDosome is not yet fully established; however, as the limited clinical brain anatomical data available for our *PIDD1* mutation families appears to echo those reported for *CRADD* mutations, this is certainly something that needs to be further explored. Future work will focus on whether *Pidd1* mice also share such features, regardless of the lack of a clear cognitive deficit. However, the phenotypic commonalities observed in humans thus far suggest that further studies into other PIDDosome pathway components as disease or risk genes for neurodevelopmental disorders are clearly warranted.

## Supplementary information

Supplementary Information
