# Supplementary material for: Biallelic mutations in the death domain of PIDD1 impair caspase-2 activation and are associated with intellectual disability
Source: Transl Psychiatry. 2021 Jan 5;11:1. doi: 10.1038/s41398-020-01158-w (PMC7791037; doi:10.1038/s41398-020-01158-w)
Supplement: Supplementary file 1 — Supplementary Information [file 41398_2020_1158_MOESM1_ESM.docx]

**Supplementary Information**

**Supplementary Figure 1: Additional western blots for PIDD1 WT versus mutant, and co-IP with CRADD:** (**A**) HEK293T cell lysate overexpressing N terminally fused GFP protein with PIDD1-WT and PIDD1-Q863* showing full length (GFP-PIDD1-FL ~27 kDa+~99kDa=~126 kDa) and cleaved PIDD1-N (27 kDa+48 kDa= ~75 kDa) anti-GFP stained SDS-PAGE gel; (**B**) anti-GFP WB of Co-IP from HEK293T cell lysate overexpressing both CRADD C-terminally tagged with DsRed and GFP-PIDD-DD-WT, GFP-PIDD1-DD-R815W and GFP-PIDD1-DD-Q863* (all N-terminally tagged with GFP). PIDD1-DD-WT-GFP: GFP-NLGDAETGFLTQSN LLSVAGRLGLDWPAVALHLGVSYREVQRIRHEFRDDLDEQIRHMLFSWAERQAGQPGAVGLLVQAL EQSDRDVAEEVRAVL =~37.51 kDa; PIDD-DD-R815W-GFP: GFP-NLGDAETGFLTQSNLLSVAGRLGLDWPAVALHLGVSYWEVQRIRHEFRDDLDQIRHMLFSWAERQAGQPGAVGLLVQAL EQSDR DVAEEVRAVL =~37.51 kDa; PIDD-DD-Q863*-GFP: NLGDAETGFLTQS NLLSVAGRLGLDWPAVALHLGVSYREVQRIRHEFRDDLDEQIRHMLFSWAERQAGQPGAVGLLVQALEQSDR =~~36.43 kDa; (**C**) CRADD (DsRed ~28 kDa+ CRADD ~22.75 kDa = 50.75 kDa) anti-DsRed WB of Co-IP from HEK293T cell lysate overexpressing both WT-CRADD C-terminally tagged with DsRed and GFP-PIDD1-DD-WT, GFP-PIDD1-DD-R815W and GFP-PIDD1-DD-Q863* (all C-terminally tagged with GFP).

**(D)** **Cell-based protein stability of mutant versus wild type PIDD1; 12 hr CHX chase experiment.** HEK293T cells were transiently transfected with vectors encoding FLAG-tagged PIDD1 wild type (WT), and DD mutants Q863*, R815W, G876S, or the splice acceptor (SA) mutant. After 24 hrs the cells were treated with cycloheximide (CHX), alone or in combination with the proteasomal inhibitor MG-132, for the times indicated, and were then processed for western blotting using and antiFLAG antibody, in a 12 hr chase experiment. A shorter, 4 hr chase experiment was also performed, and results shown in main text, Figure 4B. Reprobing with an antibody recognizing HSP90, or CHK1, was done to confirm comparable protein loading. PIDD1-FL (*), -C (§), and –CC (#) are indicated.

**
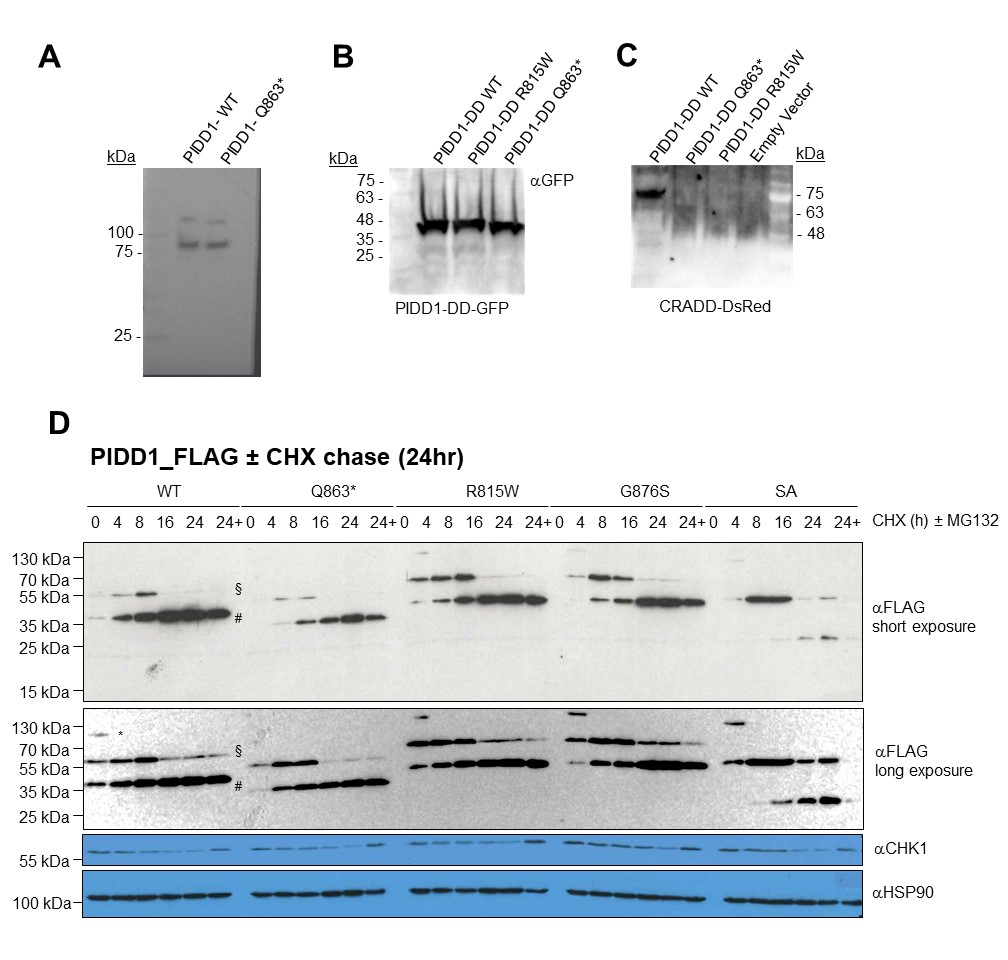
**

**Supplementary Table 1: Gateway Cloning and site-directed mutagenesis PCR primers for PIDD1 and CRADD.**

| **Primer** | **Sequence** | Total PCR product size (bp) | attB1/B2 seq | PIDD1 PCR product |
| --- | --- | --- | --- | --- |
| **Gateway Cloning Primers** | | | | |
| PIDD1-FL-attB1-F | GGGGACAAGTTTGTACAAAAAAGCAGGCTTCACCATGGCTGCAACG TG | 2535 | 65 | 2600 |
| PIDD1-FL-attB2-R | GGGGACCACTTTGTACAAGAAAGCTGGGTGGGCCTGGGCAGGCTC |  |  |  |
| PIDD1-FL-attB1-F | GGGGACAAGTTTGTACAAAAAAGCAGGCTTCACCATGGCTGCAACGGTG | 2679 | 65 | 2744 |
| PIDD1-Q863-attB2-R | GGGGACCACTTTGTACAAGAAAGCTGGGTGCCGGTCACTCTGCTC |  |  |  |
| PIDD1-FL-attB1-F | GGGGACAAGTTTGTACAAAAAAGCAGGCTTCACCATGGCTGCAACGGTG | 2679 | 65 | 2744 |
| PIDD1-FL-attB2-R | GGGG AC CAC TTT GTA CAA GAA AGC TGG GTG GGCCTGGGCAGGCTC |  |  |  |
| PIDD1-Death-F-attB1 | GGGGACAAGTTTGTACAAAAAAGCAGGCTTCATGAATCTGGGAGATGCCGAGAC | 288 | 65 | 353 |
| PIDD1-Death-R-attB2 | GGGGACCACTTTGTACAAGAAAGCTGGGTCCAAGACTGCGCGCACCTCTTC |  |  |  |
| **Site Directed Mutagenesis Primers** | | | | |
| PIDD1-Q863-SiteMut-F | GAGTGACCGGTAGGACGTGGC |  |  |  |
| PIDD1-Q863-SiteMut-R | TGCTCCAGGGCCTGCACC |  |  |  |
| PIDD1-R815W-SiteMut-F | GGGGTGTCCTACTGGGAGGTGCAGC |  |  |  |
| PIDD1-R815W -SiteMut-R | GCTGCACCTCCCAGTAGGACACCCC |  |  |  |
| **DSRed N1 monomer Cloning PCR Primers details** | | | | |
| CRADD-REDN1-F | **CGCGGGCCCGGGATCATGGAGGCCAGAGACAAAC** | 627 |  |  |
| CRADD-REDN1-R | **GGC GAC CGG TGG ATC** **CTC CAA CAT GTG CAG GAG** |  |  |  |

**Supplementary Table 2: Primers for exon trap generation and RT-PCR sequencing of “trapped” exons.** Substituted bases are underlined, and the *Sal*I recognition sequence is indicated in lower-case.

| **Primer** | **Sequence** |
| --- | --- |
| PIDD1_Exon15_SalI_F | 5’-GACTgtcgacCACTCTGCCCATCAAGCTG-3’ |
| PIDD1_Exon15_SalI_R | 5’-GACTgtcgacCCCAGGAGAAGAGCATGTGA-3 |
| PIDD1_Exon15_Mutagenesis_F | 5’-CTTTTTCCTTCCAACAAAGACTTCGAGGGTCC-3’ |
| PIDD1_Exon15_Mutagenesis_R | 5’-GGACCCTCGAAGTCTTTGTTGGAAGGAAAAAG-3’ |
| pET01_cDNA_PCR_F | 5’-ATCGATCTGCTTCCTGGC-3’ |
| pET01_cDNA_PCR_R | 5’-GGGCCACCTCCAGTGCC-3’ |

**Supplementary Table 3: Primers for generation of FLAG-tagged PIDD1 constructs**

| **Primer** | **sequence** |
| --- | --- |
| Fwd_hPIDD1_G876S | 5´gca gtc ttg gag ctc agc cgc cgc aag tac c 3´ |
| Rev_hPIDD1_G876S | 5´ggt act tgc ggc ggc tga gct cca aga ctg c 3´ |
| Fwd_hPIDD1_R815W | 5´ctg ggg gtg tcc tac tgg gag gtg cag cgc a 3´ |
| Rev_hPIDD1_R815W | 5´tgc gct gca cct ccc agt agg aca ccc cca g 3´ |
| Fwd_hPIDD1_FLAG_Q863* | 5´gagcagagtgaccgggattacaaggatgacgatgacaaataagtcttggagctcgg 3´ |
| Rev_hPIDD1_FLAG_Q863* | 5´ccgagctccaagacttatttgtcatcgtcatccttgtaatcccggtcactctgctc 3´ |
| Rev_hPIDD1_BamHI_ Δ_Exon15 | 5´ccc ctc gga ccc tcg gga tcc cgg cag ctt gat gg 3´ |
| Fwd_PAMmut hPIDD1_F | 5´gagccaccgccacacccatcaccatcc 3´ |
| Rev_PAMmut hPIDD1_R | 5´ggatggtgatgggtgtggcggtggctc 3´ |
| CMV_fwd | 5´cgc aaa tgg gcg gta ggc gtg 3´ |

**Supplementary Figure 2: Clustal Omega (www.ebi.ac.uk) alignment of PIDD1 DEATH domains orthologues from various species representative of major evolutionary families across the vertebrate phylum.**  The DEATH domain is from PDB:2of5_L (residues 778 to 883). The R815W missense and Q863* nonsense mutations are indicated (magenta), also a G876S heterozygous variant detected in an unrelated affected individual. The sequences shown are from: human: NP_665893.2; mouse: NP_073145.1; opossum: XP_016282786.1; chicken: XP_424108.3; alligator: XP_006028676.1 [Alligator sinensis]; Xenopus (Western clawed frog): XP_004913731.1 [Xenopus (Silurana) tropicalis]; zebrafish: E9QFQ1_DANRE. Residues with identity across all seven species are shaded grey.

**
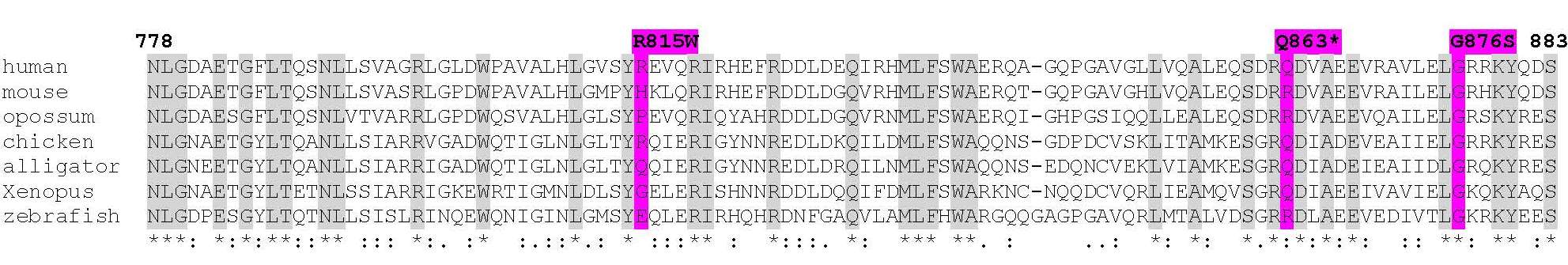
**

**Supplementary Figure 3: Electropherograms (Sanger sequencing) of variant c.1909C>T in *PIDD1*** **in family Manipal-1**: in homozygous state in (i) proband (II-3), and heterozygous state in (ii) elder sibling (II-2) (iii) mother (I-2) and (iv) father (I-1). The pedigree is shown in the main text, Figure 1.


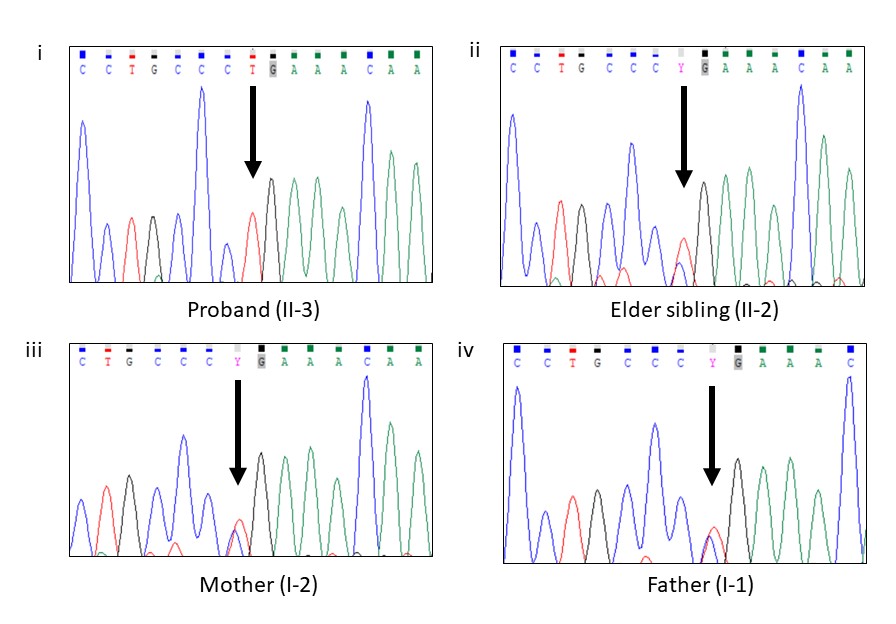


**Clinical Observations:**

**Pakistani Family AS110 (Gln863*):**

**AS110 IV-3:** Male, 30 yrs. old at time of examination, has a history of epilepsy (generalized tonic-clonic seizures). His behavior is described as odd, generally calm, with intermittent aggression, unable to learn, no history of antisocial behavior (undressing, micturating, defecating publically), eats/drinks normally, no self-harm or hurting others. No dysmorphic features. Ophthalmological analysis indicated no squint, normal fundus and retina, and no cataract. **MRI with IV contrast:** multiecho, with multiplanar images were obtained, also post-gadolinium; normal anatomy and blood flow. Subtle lissencephaly of the cortical layers of the cerebral hemispheres was noted, most pronounced in bilateral frontal lobes. **Neurological report:** cranial nerves normal; lower limbs: tone normal, power 5/5, reflexes ++, plantars withdrawal bilaterally; cerebellum normal; gait- reduced arm swing, bradykinesia. **Psychiatry and IQ:** His milestones were delayed. He requires direction for self-care. He did not understand examiners commands to engage in testing. The level of intellectual disability is severe to profound.

**AS110 IV-1:** Male, 18 yrs. old at time of examination. Issues with learning identified since childhood. Relatives do not recall milestones. He shows inappropriate behaviors, and self-injurious behavior occasionally; No dysmorphic features. **Ophthalmological analysis:** no cataract, no squint, normal fundus but occasional white flecks seen distributed throughout, retinoscopy-neutral reflex, no errors of reflection; **Neurological report:** cranial nerves normal, upper limb- tone normal; reflexes normal ++; power 5/5; lower limb- tone normal, reflexes normal ++; power 5/5; L and R plantars down. Cerebellum normal; gait: reduced arm swing, bradykinesia. **Psychiatry and IQ:** Intellectual disability; is dependent on others for day-to-day activities. Tested using Standard Progressive Matrices, scored “8”, putting him at 5^th^ centile.

**Pakistani Family AS105 (Gln863*):**

**AS105 III-3:** Male, 5 yrs. old at time of examination, has delayed milestones, with apparent regression after 3 years, and with difficulty learning new things. **Ophthalmological analysis:** Right, 30% esotropia; no nystagmus, fundus/discs normal. **MRI with IV contrast:** multiecho, multiplanar images obtained, also post-gadolinium; normal anatomy and blood flow. Mild decreased volume of the body and splenium of the corpus callosum was noted. **Neurological report:** cranial nerves restricted eye movement on horizontal gaze, bilaterally; upper limb- tone normal; reflexes normal ++; power 5/5; lower limb- tone normal, reflexes normal ++; power 5/5; gait normal. **Psychiatry and IQ:** On history and examination, he fulfills criteria for Attention Deficit Hyperactivity Disorder (ADHD). He has history of physical aggression to others. It was not possible to engage him in formal testing because of his hyperactivity and inattention. His estimated level of intellectual disability was in the severe range based on the history and information through informal psychological assessment.

**AS105 III-2:** Female, 8 yrs. old at time of examination, has a history of delayed milestones, difficulty learning, attention deficit, hyperactivity and aggressive behavior, convergent squint, bite marks on hands. **Neurological report:** upper limb- tone normal; reflexes normal ++; power 5/5; lower limb- tone normal, reflexes normal ++; power 5/5; L and R plantars downgoing; gait normal; **Psychiatry and IQ:** She meets the criteria for ADHD. She is dependent on her family for toileting and self-care. It was difficult to engage her in testing.. WISC-R scores: scored 6 on verbal testing, 0 on performance testing. These low scores are partially because of lack of engagement in testing. She has severe to profound intellectual disability.

**AS105 III- DNA not available (genotype for mutations not confirmed):** Male, 20 yrs. old at time of examination, with a history of delayed milestones; seizures started at 1yr, continued to 10yrs, now epilepsy free. Delayed learning. **Neurological report:** upper limb- tone normal; reflexes normal ++; power 5/5, sensations intact; lower limb-tone normal, reflexes normal ++; power 5/5; sensations intact. Plantars downgoing. Normal gait. **Psychiatry and IQ:** His mile stones were delayed. He required help with self-care till age 15. He has always had difficulty with controlling anger and has been physically and verbally aggressive to others. He has a history of self-injury. The Wechsler Adult Intelligence Scale (WAIS) scores: verbal=7; performance= 12; may not be true reflection of his ability as he did not engage with testing. He however has severe to profound intellectual disability.

**Iranian Family M278 (Adapted from Hu et al., 2019) c.2275-1G>A; Arg759Glyfs*1**

Two affected brothers from healthy parents from Hormozgan province in southern Iran. II:1 (M) and II:2 (M) were both born at term after uneventful pregnancies, neonatal periods and normal development. They developed head control at about 4 months, started to sit at 8 months, to stand at 14 months, to speak single words at 12 months, and to walk at 18 months. They both developed seizures, beginning at age 12 and 14 years, respectively, which were controlled through medication. Their vision and hearing were normal. They had a kind and quiet temperament, and no aggressive behavior reported. At 21 and 18 years, their heights were 178 (50%ile) and 167 cm (10%ile), respectively, and occipitofrontal circumferences (OFC) were 56 cm (0SD) and 54 cm (–2.5SD). Their facial appearance was considered normal, other than thick lips. They had normal gait, and normal speech development. Their cognitive status was evaluated using WAIS-IV, and showed IQs of 50 and 55, respectively, in the range of moderate ID.

**Iranian Family M8700004** **(Adapted from Hu et al., 2019); c.2443C>T; Arg815Trp**

Three affected siblings from healthy first cousin parents from Fars province in central Iran.III:3 (F), III:5 (F), and III:6 (M) were born at full term with normal birth weight, height and OFC (not documented). Psychomotor delay was reported by their mother. All three affected siblings developed generalized seizures during infancy, which were controlled by medication. Maternal report suggested features of psychosis, poor memory, and sleep disturbances. They were unable to count money, memorize family names, or look after personal hygiene. Their hearing and vision were reported as normal. At 26, 24, and 23 years of age, their heights were 150 (3%ile), 144 (<3%ile), and 168 cm (25%ile), respectively, and OFCs were 53 (–2SD), 56 (–1SD), and 54 cm (–2SD). They had normal facial appearance. Eye-to-eye contact with family members was evident, however social communication was poor, and difficulties with concentrating. They had a normal gait and had slow, slurred speech with fewer than 10 words. Their cognitive status was evaluated using WAIS-IV, and showed IQs of 24 (III:3), 30 (III:5), and 33 (III:6), respectively, in the range of severe ID. Evaluation by a psychiatrist showed psychosis in all three patients.

**Indian Family, Manipal-1; c.1909C>T; p.Arg637***

A ten-year-old female, was ascertained, second child born to a consanguineously married couple. She was born at term via normal vaginal delivery. Her birth weight was 2.5 kg (-1.8 SD). She started independent sitting by eight months of age and independent walking by 14 months. Her language milestones were delayed. She started cooing and babbling only by 19months, spoke monosyllables by 24 months and bi syllables by 26 months. Currently, she has poor scholastic performance and learning disabilities. No significant behavioral abnormality present, except persistent nail biting. She had one episode of seizure at four years of age. She underwent surgery at two years of age for fused labia and for renal calculus at five years of age. On examination, her head circumference was 50 cm (-2.8 SD), height was 122 cm (-1.8 SD) and weight was 21.5 kg (1.6 SD). She had bite marks on fingernails/nail beds. She underwent intellectual quotient (IQ) testing by Seguin Form Board Test at four years and four months of age and diagnosed with mild mental retardation with an IQ of 68. At eight years of age, repeat IQ testing revealed a score of 75. Vanderbilt Assessment Scale for ADHD, also at age 8 yrs, showed that she had features of both inattention and hyperactivity. Brain magnetic resonance imaging (MRI) was done at eight years of age (see Figure 1F-H). It showed broad and smooth gyri in bilateral frontal and temporal lobes with reduced sulcations suggestive of pachygyria. The corpus callosum was noted to be short and thick. An electroencephalogram (EEG) was done at nine years of age and it showed generalized epileptiform activities with photoparoxysmal response (PPR).

She has a 17-years-old male sibling, born at term via full term normal vaginal delivery. His birth weight was 2.5 kg (1.2 SD) and he cried immediately at birth. There was no history of neonatal ICU admission or any other co-morbidities at the time of birth. His developmental milestones were age appropriate according to the parents. He had two episodes of seizures at 14 years of age in a span of two months for which he is under medication. On examination, his height was 176.5 cm (0.16 SD), his weight was 63 kg (1.8 SD) and head circumference was 54.3 cm (-1.4 SD). He had excessive nail-biting habit with bite marks on fingers and toes. He also has a café au lait spot (10x7cm) on anterior chest. Hyperpigmentation was also noted on both the arms. He underwent IQ testing and cognitive assessment battery for ADHD. His IQ was noted to be 104 with average level of intelligence while Vanderbilt Assessment Scale for ADHD showed no major behavioral issues. MRI was done for him at 14 years of age and showed no significant abnormalities. EEG was done for him at 14 years of age and it showed generalized epileptiform discharges and occasional right focal discharges.

**Supplementary Figure 4:** Outline of strategy for, and results of exon trapping. **A.** Genomic organization flanking splice acceptor mutation c.2275-1G>A. An amplicon encompassing part of exon 14, intron 14, exon 15, intron 15 and part of the final exon, exon 16, was PCR amplified (631 bp) from wild type DNA and **B.** cloned into the *Sal*I restriction site within the multiple cloning site of pET01 vector (MoBiTec GmbH). **C.** Site directed mutagenesis was used to generate the c.2275-1G>A substitution. **D.** Following transfection into HEK293 cells, RT-PCR amplification across the insert was performed for i. pET01 vector with no insert, ii. pET01 vector with the WT insert, and iii. pET01 vector with the c.2275-1G>A mutation. **E.** Sanger sequencing of the RT-PCR product indicated a loss of exon 15 from the transcript for the mutant construct only.

**
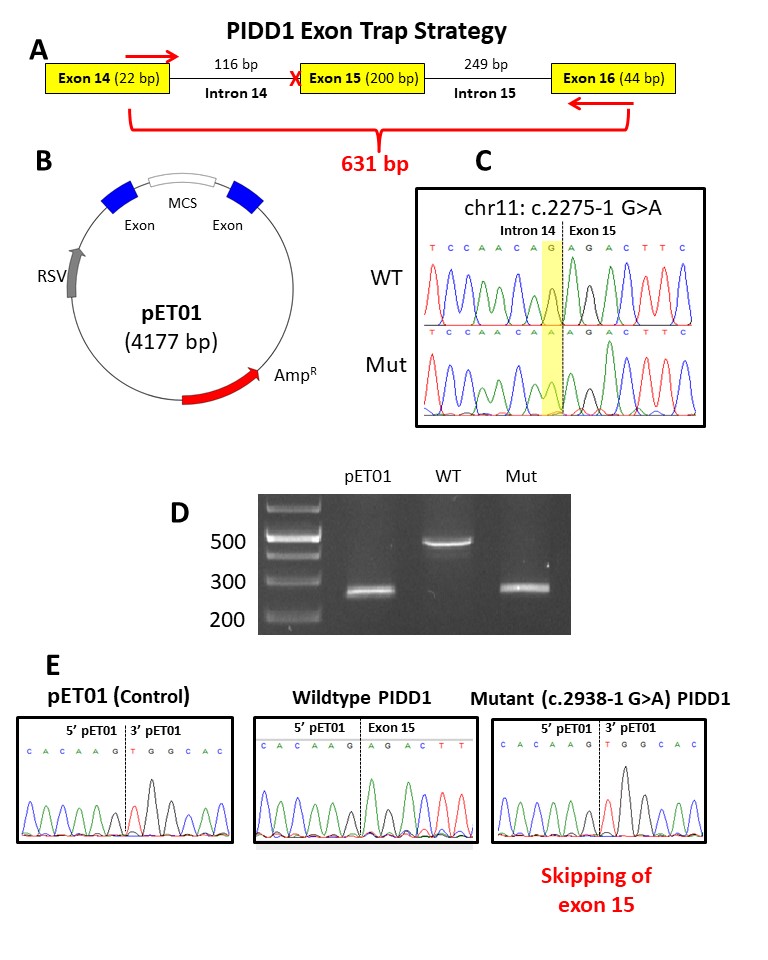
**

**Supplementary Figure 5. Additional *Pidd1* KO versus WT mouse testing:** tests shown were performed on 3-month-old male mice (WT: N=10; KO: N=9). Mann-Whitney U test indicated no significant difference between groups.

##### Supplementary Figure 6a. *PIDD1*: gene expression in adult human tissues (<https://www.gtexportal.org/>). GTEx Analysis Release V8 (dbGaP Accession phs000424.v8.p2). Expression values are shown as transcripts per million (TPM) on the y axis, with alternatively splice transcripts collapsed to a single gene. Box plots are shown as median and 25^th^ and 75^th^ percentiles. A linear scale was used, with ‘outliers’ off. The Genotype-Tissue Expression (GTEx) Project was supported by the [Common Fund](https://commonfund.nih.gov/GTEx) of the Office of the Director of the National Institutes of Health, and by NCI, NHGRI, NHLBI, NIDA, NIMH, and NINDS. The data used for the analyses described in this manuscript were obtained from the GTEx Portal on 20^th^ Nov 2020.


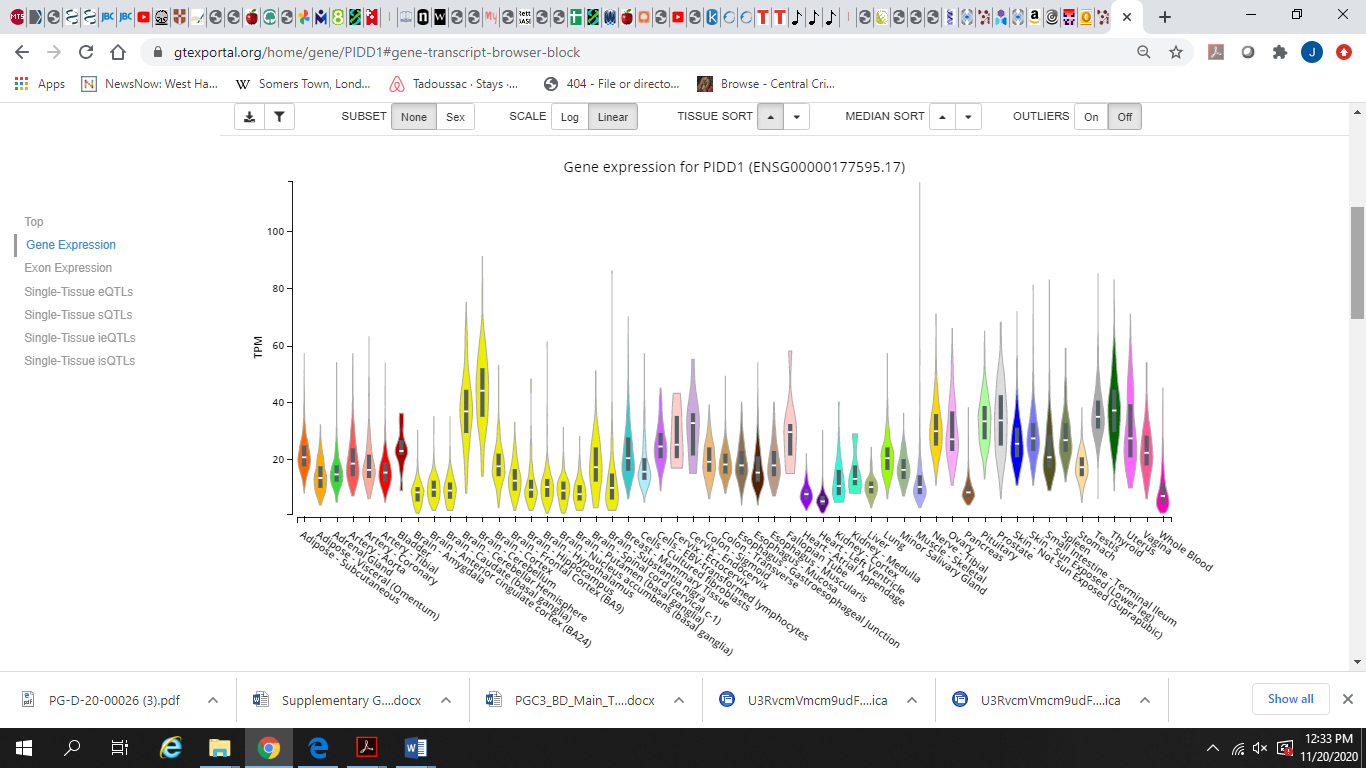


**Supplementary Figure 7a Expression of *PIDD1* in BrainSpan human data in 4 regions by age.** Expression analysis was performed on the exon microarray datasets, which show higher expression of *PIDD1* in the early developmental stages and specifically in the hippocampus formation between 8- and 12 post conception weeks (pcw). The original dataset was from Miller et al, 2014.


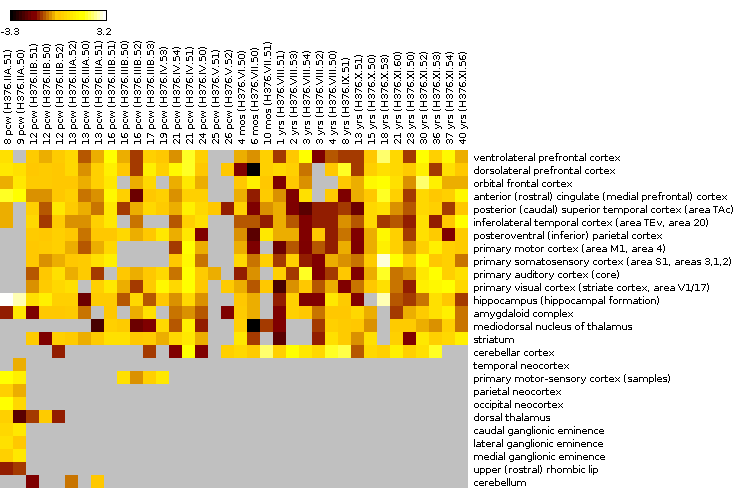


**Supplementary Figure 7b*:* Expression of *PIDD1* in BrainSpan data of 4 regions by age.** Expression analysis was performed on the RNAseq datasets which show higher expression of *PIDD1* between 13- and 19- weeks post conception. Regions with high expression were posteroventral inferior cortex, primary visual cortex and primary motor-sensory cortex. The RNAseq data normalization seems to have more artefacts than the exon array especially considering the few genes that being used which is confounding the signal. The original dataset was from Miller et al, 2014.


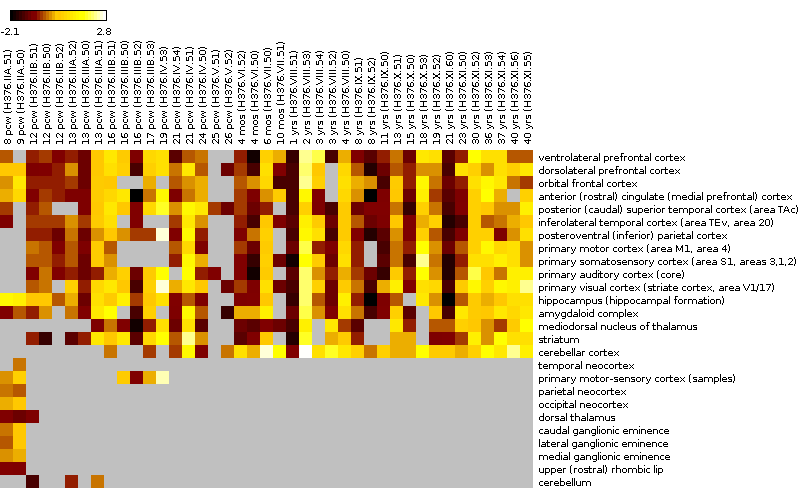


**Supplementary Figure 7c Expression of *PIDD1* + *CRADD* + *CASP2* in BrainSpan human data in 4 regions by age.** Expression analysis was performed on the exon microarray datasets, which show higher expression of PIDD1 in the early developmental stages and specifically in the hippocampus formation between 8- and 12-weeks post-conception. The original dataset was from Miller et al, 2014.


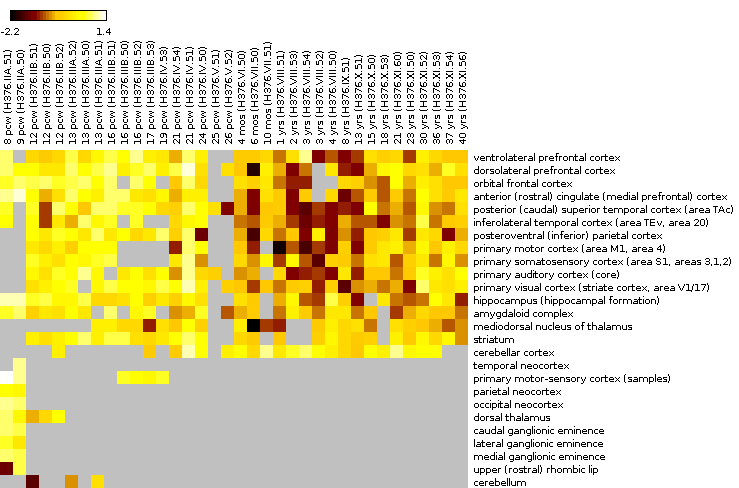


**Supplementary Figure 7d:** Gene expression ***PIDD1* + *CRADD* + *CASP2*** in the BrainSpan RNAseq data from four regions by age. High expression is seen between 13 and 19 weeks post-conception, however significant normalization artefacts exist and confound the expression profiling, as with the RNAseq data for just *PIDD1*. The original dataset was from Miller et al, 2014.


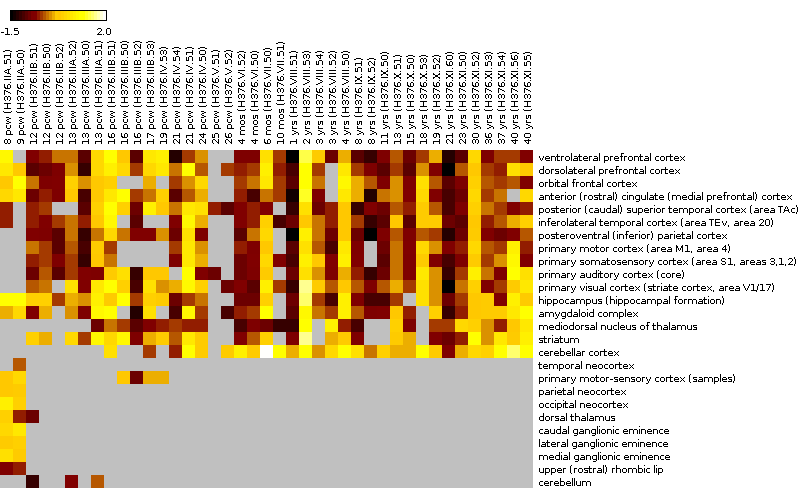


**Supplementary Figure 7e.** RNAseq expression profiles by brain region and developmental stages using the Human PsychEncode expression dataset (Zhu et al, 2018), revealing co-expresssion for *PIDD1*, *CRADD* and *CASP2* in the cortex at 8-9 pcw, and between 12 and 16 weeks in the hippocampus and amygdaloid complex, and in striatum between12 pcw and 4 months, and in cerebellar cortex from ~13 pcw onwards.
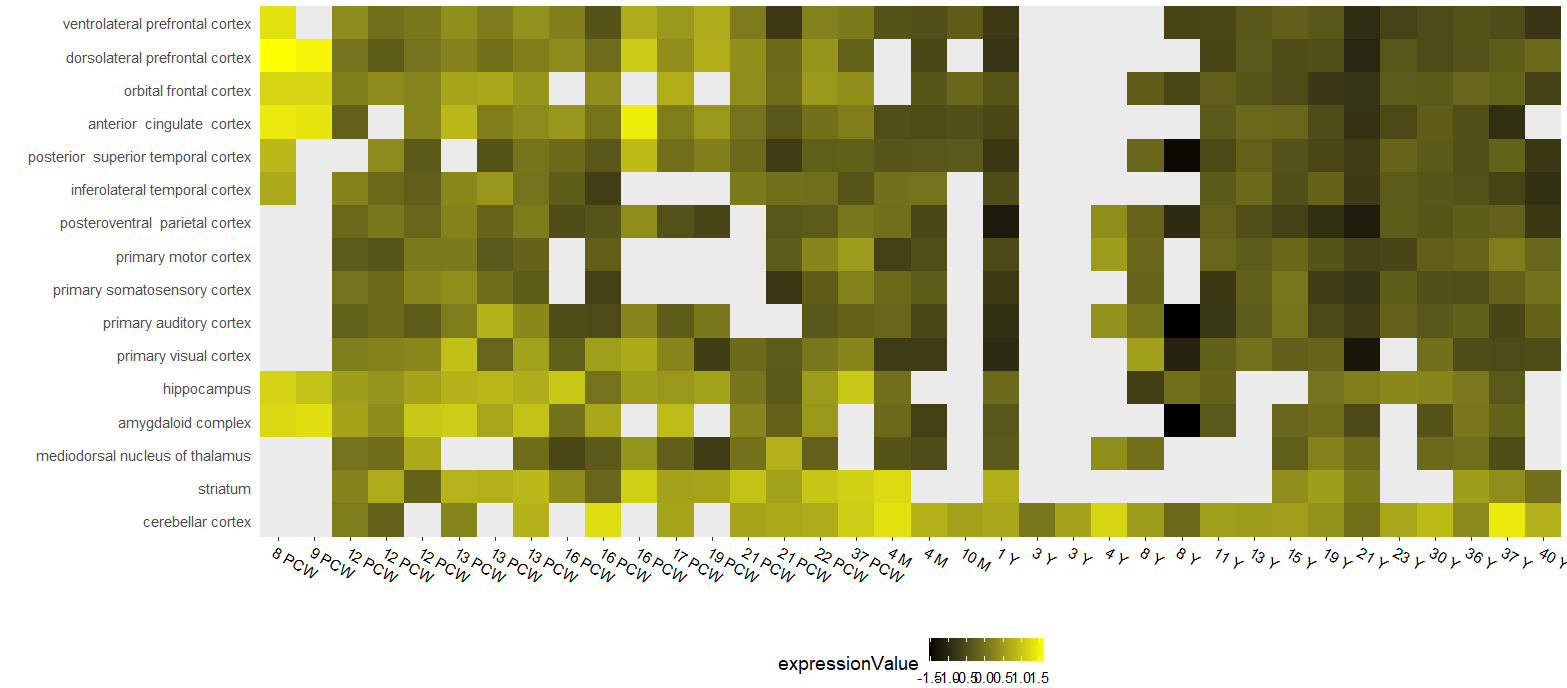


**Supplementary Figure 7f:** Gene expression of *PIDD1* plus related genes (Gene set P: *PIDD1*, *CRADD*, *CASP2*, *MADD*, *FADD*) and other lissencephaly genes (Gene set L: *RELN*, *TUBA1A*, *NDE1*, *KATNB1*, *CDK5*, *ARX*, *DCX*, *LPHN1*, *LPHN2*, *LPHN3*) in the BrainSpan Exon array dataset, for 4 regions by time. The figure was created with additional genes related to PIDD1 and lissencephaly. Expression is clearer and shows overall higher levels of expression in the early stages of development between 8 weeks post conception to four months after birth. There is higher expression in the striatum which has been linked to information integration in Autism Spectrum Disorder. Expression in areas such as the hippocampus and inferolateral temporal cortex are also elevated. Data is more consistent with additional gene expression with better resolution. The original dataset was from Miller et al, 2014

LPH
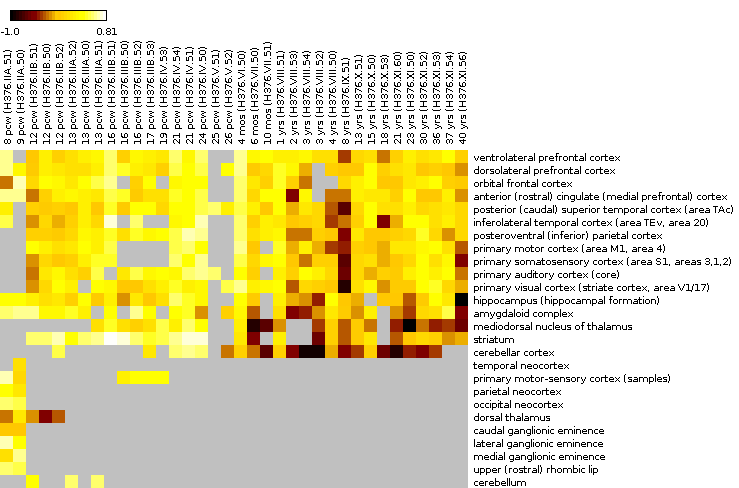


**Supplementary Figure 7g:** Gene expression of P+L gene set in the BrainSpan RNAseq data from four regions by age. Additional genes allowed for clearer and more consistent expression to be interrogated with less artefactual confounders from the normalization method. Striatum, inferolateral temporal cortex and ventrolateral prefrontal cortex. This RNAseq expression profile is more consistent with the exon array from just the *PIDD1* expression profile (Supplementary Figure 7b) and the P+L genes (Supplementary Figure 7e). The original dataset was from Miller et al, 2014.


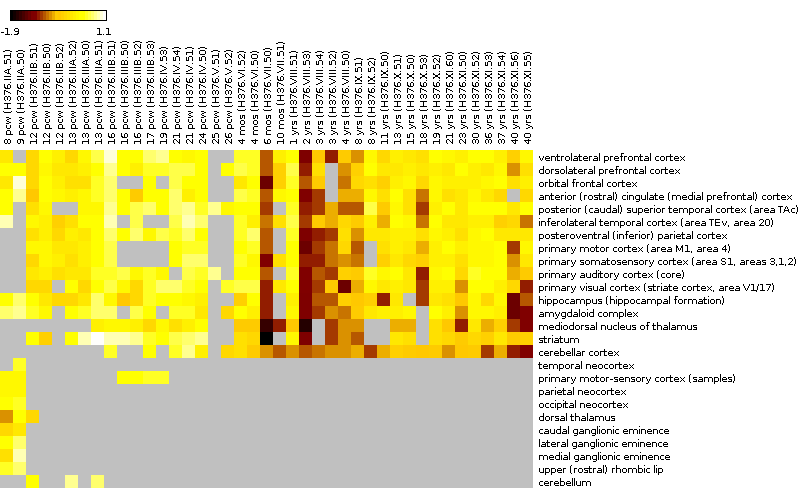


**Supplementary Figure 7h:** RNAseq expression profiles by brain region and developmental stages using the Human PsychEncode expression dataset (Zhu et al, 2018), revealing highest co-expresssion for P+L genes between 13 pcw and 21 pcw, particularly in the striatum but overall elevated across all regions.


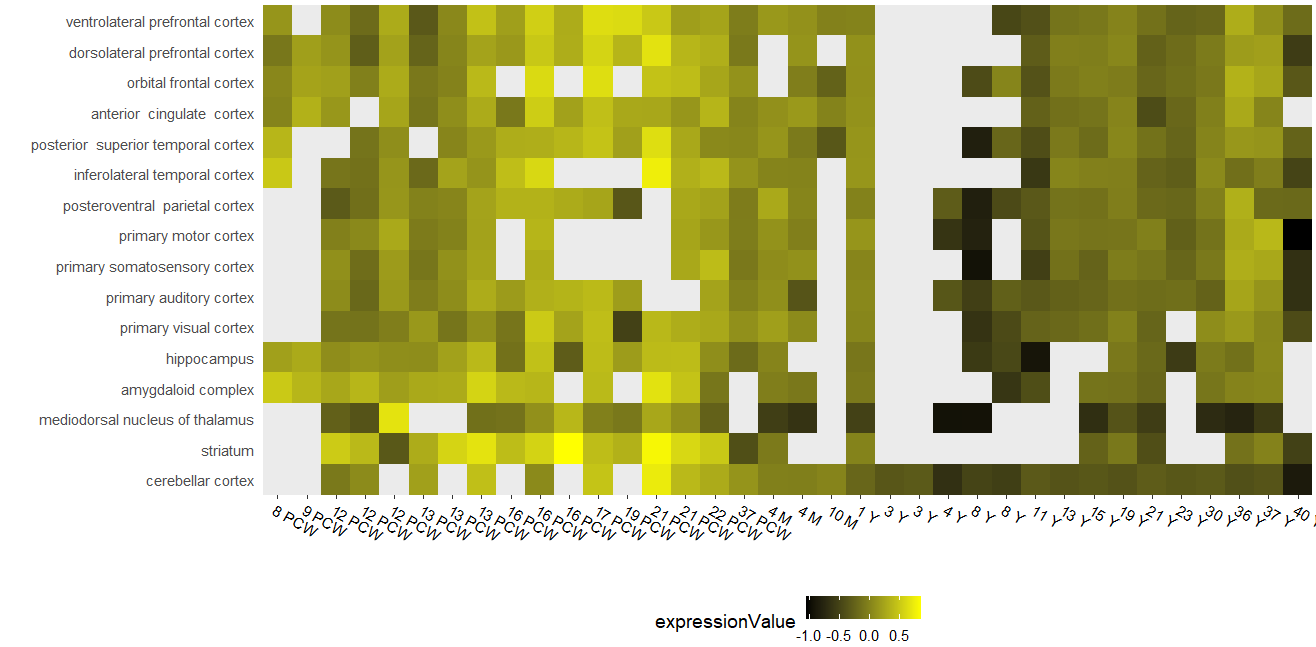


**Supplementary Figure 7i:** Probability of average expression of P+L gene set from the BrainSpan RNAseq data for 4 regions by time adjusted for multiple testing correction. The figure shows 13 to 16 weeks post conception expression is approaching significance in the striatum region for these genes. Additional genes related to PIDD1 may increase power and trend towards being significant after multiple testing correction.


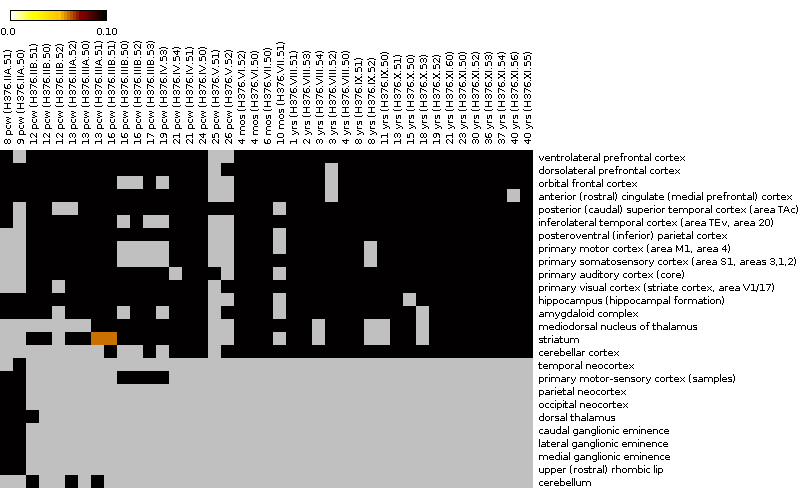


**Supplementary Figure 8: Expression of 6 Adult Brains for a) *PIDD1* only; b) *PIDD1*+*CRADD*+*CASP2*; c) P+L gene set.** a) shows higher expression of *PIDD1* in the corpus callosum (CC), occipital lobe (OL) and cerebellum, with low expression in the hindbrain and brainstem; b) heightened expression in the CC, cerebellum and thalamus (TH), but less expression in the frontal, parietal, temporal and occipital lobes (FL, PL, TL and OL) than for *PIDD1* alone; c) shows higher expression in FL, PL, TL and OL, but low in CC and other regions. Original dataset (two brains) from Hawrylycz et al, 2012, with four more brains added to the dataset post-publication. Area under the curve analysis performed using <https://hbaset.msl.ubc.ca/>, with methods as described previously (Howard et al, 2020).

| a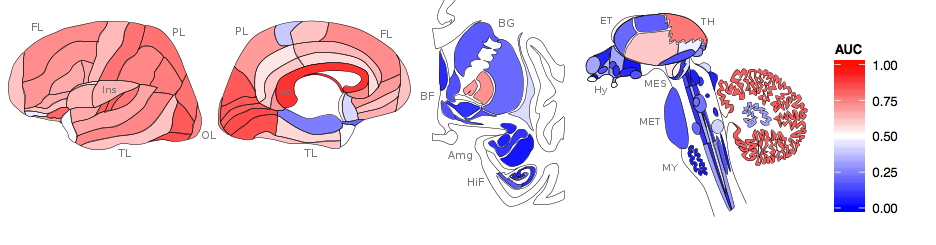 |
| --- |
| b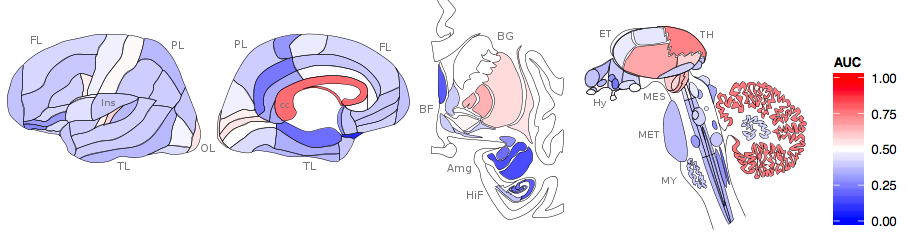 |
| c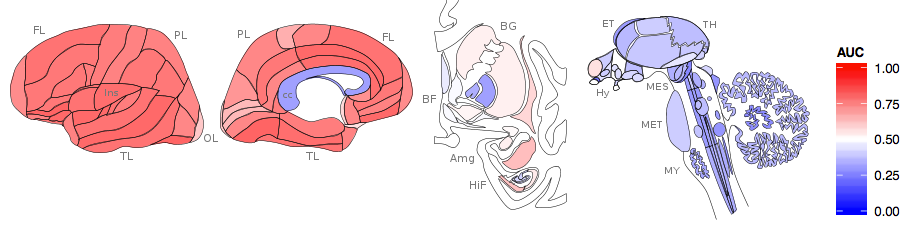 |

**Supplementary Figure 9a:** Mouse fetal single-cell RNAseq transcriptional map (La Manno et al, [eprint: <https://www.biorxiv.org/content/10.1101/2020.07.02.184051v1>]; www.mousebrain.org/development), using 161 embryos between E7 and E18.5: Reference map.


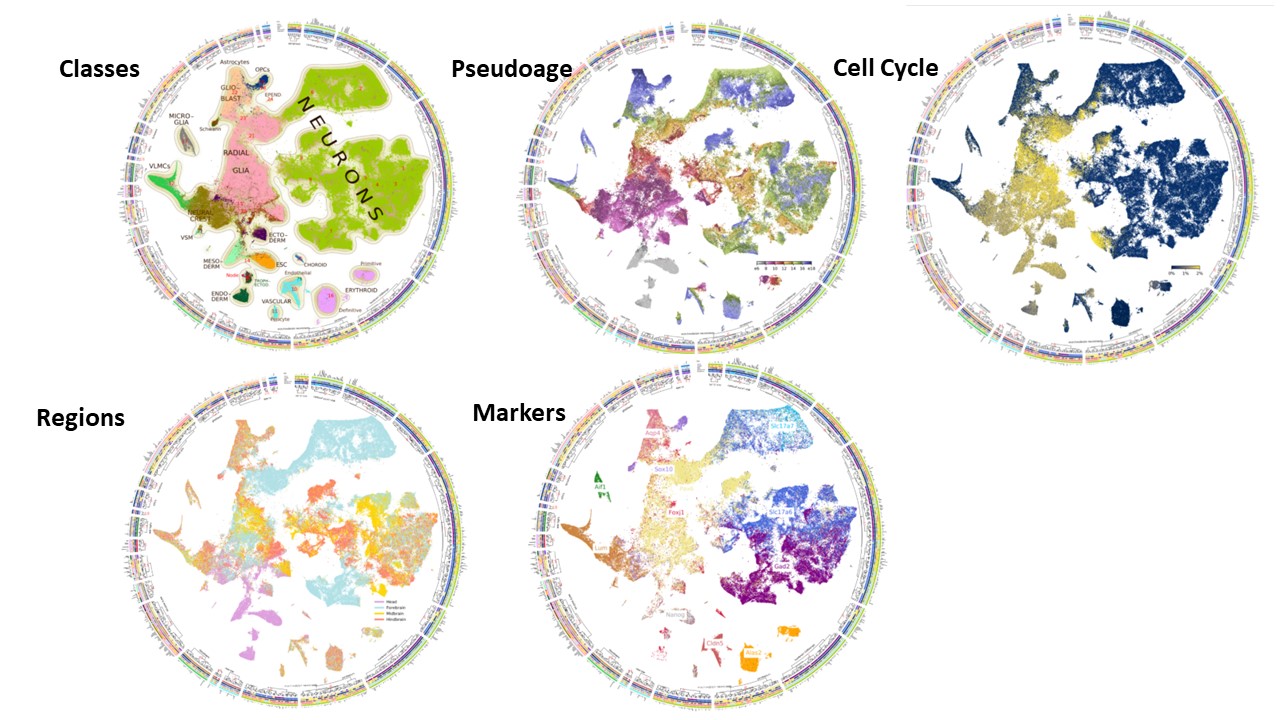


**Supplementary Figure 9b:** Mouse fetal single-cell RNAseq transcriptional map showing temporal/spatial expression of *Pidd1* (La Manno et al, [eprint: <https://www.biorxiv.org/content/10.1101/2020.07.02.184051v1>]; www.mousebrain.org/development), using 161 embryos between E7 and E18.5.


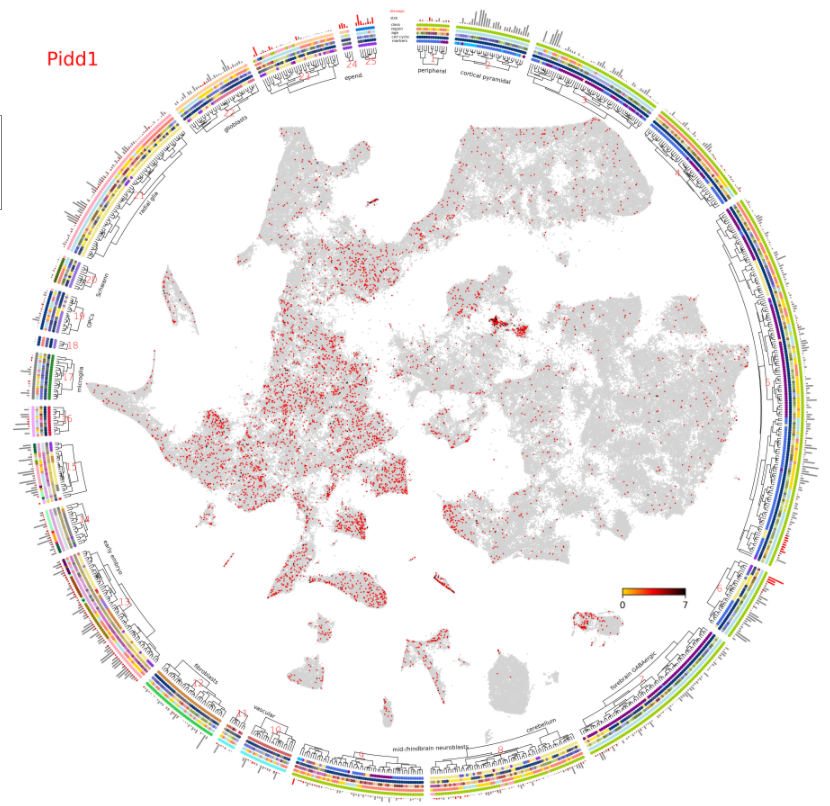


**Supplementary Figure 9c:** Mouse fetal single-cell RNAseq transcriptional map showing temporal/spatial expression of *Cradd* (La Manno et al, [eprint: <https://www.biorxiv.org/content/10.1101/2020.07.02.184051v1>]; www.mousebrain.org/development), using 161 embryos between E7 and E18.5.


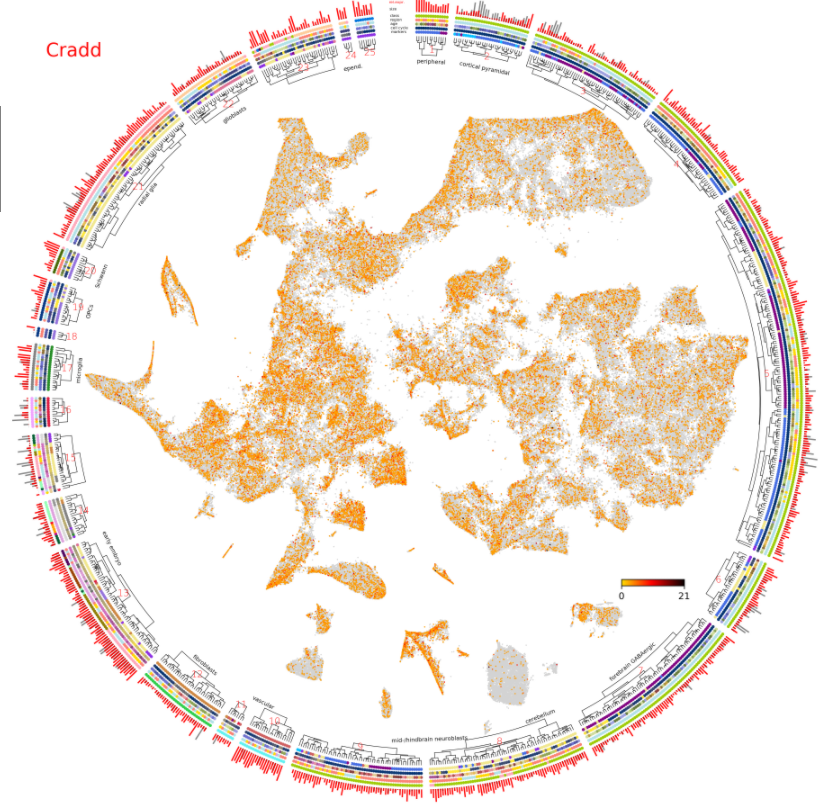


**Supplementary Figure 9d:** Mouse fetal single-cell RNAseq transcriptional map showing temporal/spatial expression of *Caspase-2* (*Casp2*) (La Manno et al, [eprint: <https://www.biorxiv.org/content/10.1101/2020.07.02.184051v1>]; www.mousebrain.org/development), using 161 embryos between E7 and E18.5.


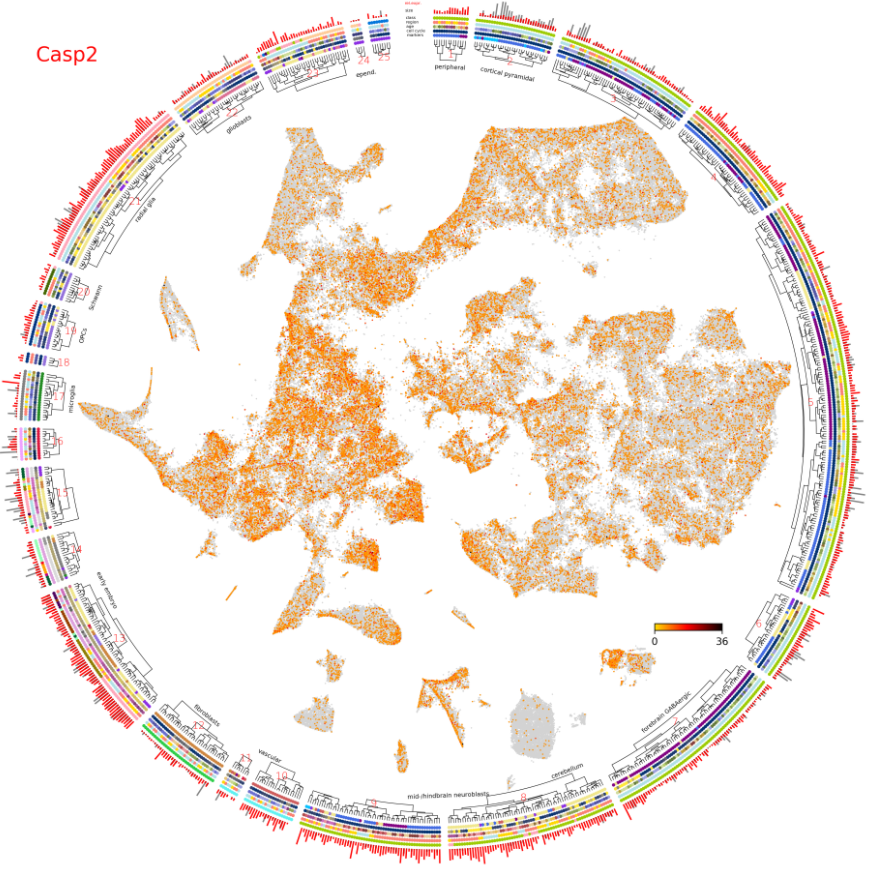


**Supplementary Table 4:** Top (p<0.05) Cell Types for Mouse Cell Typing Expression for co-expression of Pidd1, Casp2, and Cradd. Cell types/regions are ranked by Area Under the Curve (AUC). Note p-values are uncorrected for multiple testing. The original data was from Zeisel et al, 2018 [27], which used male and female mice age P12-30, as well as 6 and 8 weeks old.

| Cluster ID | Description | AUC | P Value |
| --- | --- | --- | --- |
| SYCHO1 | Cholinergic neurons, sympathetic | 0.885812 | 0.020633 |
| ENTG5 | Enteric glia | 0.881661 | 0.022037 |
| DGNBL1 | Granule neuroblasts, dentate gyrus | 0.859448 | 0.031048 |
| ENTG1 | Enteric glia, proliferating | 0.851872 | 0.034771 |
| ENTG3 | Enteric glia | 0.848363 | 0.036622 |
| ENT8 | Intrinsic sensory neurons, enteric | 0.846751 | 0.037499 |
